# Systematic review of Mendelian randomization studies on risk of cancer

**DOI:** 10.1186/s12916-022-02246-y

**Published:** 2022-02-02

**Authors:** Georgios Markozannes, Afroditi Kanellopoulou, Olympia Dimopoulou, Dimitrios Kosmidis, Xiaomeng Zhang, Lijuan Wang, Evropi Theodoratou, Dipender Gill, Stephen Burgess, Konstantinos K. Tsilidis

**Affiliations:** 1 Department of Hygiene and Epidemiology, University of Ioannina School of Medicine, Ioannina, Greece; 2 Department of Epidemiology and Biostatistics, St. Mary’s Campus, School of Public Health, Imperial College London, Norfolk Place, London, W2 1PG UK; 3 Bristol Medical School, University of Bristol, Bristol, UK; 4 Department of Hygiene, Epidemiology and Medical Statistics, Medical School, National and Kapodistrian University of Athens, Athens, Greece; 5 Centre for Global Health, Usher Institute, The University of Edinburgh, Edinburgh, UK; 6 CRUK Edinburgh Centre, Institute of Genetics and Cancer, The University of Edinburgh, Edinburgh, UK; 7 Medical Research Council Biostatistics Unit, University of Cambridge, Cambridge, UK; 8 Cardiovascular Epidemiology Unit, University of Cambridge, Cambridge, UK

**Keywords:** Mendelian randomization, Cancer, Risk factors, Systematic review, Evidence grading

## Abstract

**Background:**

We aimed to map and describe the current state of Mendelian randomization (MR) literature on cancer risk and to identify associations supported by robust evidence.

**Methods:**

We searched PubMed and Scopus up to 06/10/2020 for MR studies investigating the association of any genetically predicted risk factor with cancer risk. We categorized the reported associations based on a priori designed levels of evidence supporting a causal association into four categories, namely *robust*, *probable*, *suggestive*, and *insufficient*, based on the significance and concordance of the main MR analysis results and at least one of the MR-Egger, weighed median, MRPRESSO, and multivariable MR analyses. Associations not presenting any of the aforementioned sensitivity analyses were not graded.

**Results:**

We included 190 publications reporting on 4667 MR analyses. Most analyses (3200; 68.6%) were not accompanied by any of the assessed sensitivity analyses. Of the 1467 evaluable analyses, 87 (5.9%) were supported by *robust*, 275 (18.7%) by *probable*, and 89 (6.1%) by *suggestive* evidence. The most prominent *robust* associations were observed for anthropometric indices with risk of breast, kidney, and endometrial cancers; circulating telomere length with risk of kidney, lung, osteosarcoma, skin, thyroid, and hematological cancers; sex steroid hormones and risk of breast and endometrial cancer; and lipids with risk of breast, endometrial, and ovarian cancer.

**Conclusions:**

Despite the large amount of research on genetically predicted risk factors for cancer risk, limited associations are supported by robust evidence for causality. Most associations did not present a MR sensitivity analysis and were thus non-evaluable. Future research should focus on more thorough assessment of sensitivity MR analyses and on more transparent reporting.

**Supplementary Information:**

The online version contains supplementary material available at 10.1186/s12916-022-02246-y.

## Background

With a global burden of 18.1 million new cases and 9.9 million deaths in 2020 [1], cancer is one of the leading non-communicable diseases. Despite the extensive research in the field, a causal relationship with cancer has been established only for a limited number of risk factors. Identification of causal relationships with specific risk factors and separation from spurious associations is key to cancer prevention. Despite being considered the gold standard for identification of causal relationships, randomized controlled trials (RCT) are often impractical or even unfeasible to perform due to time constraints and ethical issues. Conversely, the capacity of epidemiological observational studies to identify causal relationships is limited, due to confounding, reverse causation, and other biases [2].

Mendelian randomization (MR) is an analytic approach which utilizes genetic variation as a randomized instrument of the exposure of interest to provide insights into causality. As genetic variants are assumed to be randomly distributed at conception, MR can be considered akin to a “natural” RCT [3, 4]. By using genetic variants (single-nucleotide polymorphisms [SNPs]) as instrumental variables (IV) to assess the association of a genetically predicted exposure with the outcome of interest, MR analyses can provide estimates less prone to some common epidemiological biases. Nevertheless, for a MR analysis to be valid, three assumptions for IVs must be met: (a) the genetic variants should be associated with the exposure; (b) the genetic variants must not be associated with measured or unmeasured confounders of the exposure-outcome association; (c) conditional on the exposure and the confounders, the genetic variants must be independent of the outcome. Given the growing availability of large-scale genomic information from published genome-wide association studies (GWAS), it is no wonder that during the past decade MR analyses have seen a substantial increase, especially after the introduction of the “two-sample” summary-data MR approach that can improve feasibility and efficiency [5].

Researchers are faced with the challenge of evaluating the MR evidence, filtering this information and deriving valid inferences. The continuously increasing amount of new scientific information coupled with the fact that two of the three MR assumptions (b and c) cannot be confirmed empirically further complicates this cumbersome task. Furthermore, the field of evaluating MR associations is rapidly evolving [6, 7]. The investigation and assessment of the potential violations of the MR assumptions, especially in the case of multiple instruments, is a key step towards a valid inference and a robust interpretation of potential causal associations. Several sensitivity analyses have been proposed that address the validity of these assumptions, and the results from MR studies that do not use them should be viewed as incomplete [8].

In this paper, we systematically reviewed the literature investigating associations between genetically predicted risk factors and any type of cancer using MR approaches. Firstly, we aimed to map and describe the current state of MR literature on cancer risk, identify areas where research has focused, and identify possible gaps and emerging areas of interest. Furthermore, we aimed to evaluate these associations using a breadth of well-established MR methods and the most commonly applied sensitivity analyses to identify those presenting robust evidence for causality. We note that the word “robust” refers to evidence of causality for the studied associations, not the quality of the analysis.

## Methods

This systematic review was conducted in accordance to the published protocol that was registered in the open Science Network registries (https://osf.io/2ruct) and is reported following the Preferred Reporting Items for Systematic Review and Meta-Analysis (PRISMA) checklist [9].

### Search Strategy

A detailed description of the search strategy and inclusion and exclusion criteria along with the data extraction process is provided in the Additional file 1: Supplementary methods [10–26]. Briefly, we searched the Medline (via PubMed) and Scopus databases from inception to 06/10/2020 using a combination of the terms “Mendelian randomization,” “genetic instrument,” and “cancer” and their synonyms for MR studies investigating the association of genetically predicted risk factors with risk of cancer development or mortality. We also screened the references of relevant reviews and the references of the included studies. We extracted information on the exposure and outcome of interest, the genetic instrument, the MR design (one-sample or two-sample, based on whether the gene-exposure and gene-outcome associations were estimated on the same or different populations), and main MR analysis results (as defined by the authors). We further extracted information on a number of sensitivity MR methods, namely MR-Egger, weighted median (WM), MRPRESSO, and also multivariable MR (MVMR).

### Evaluation of Robustness in the identified associations

**Fig. 1:**
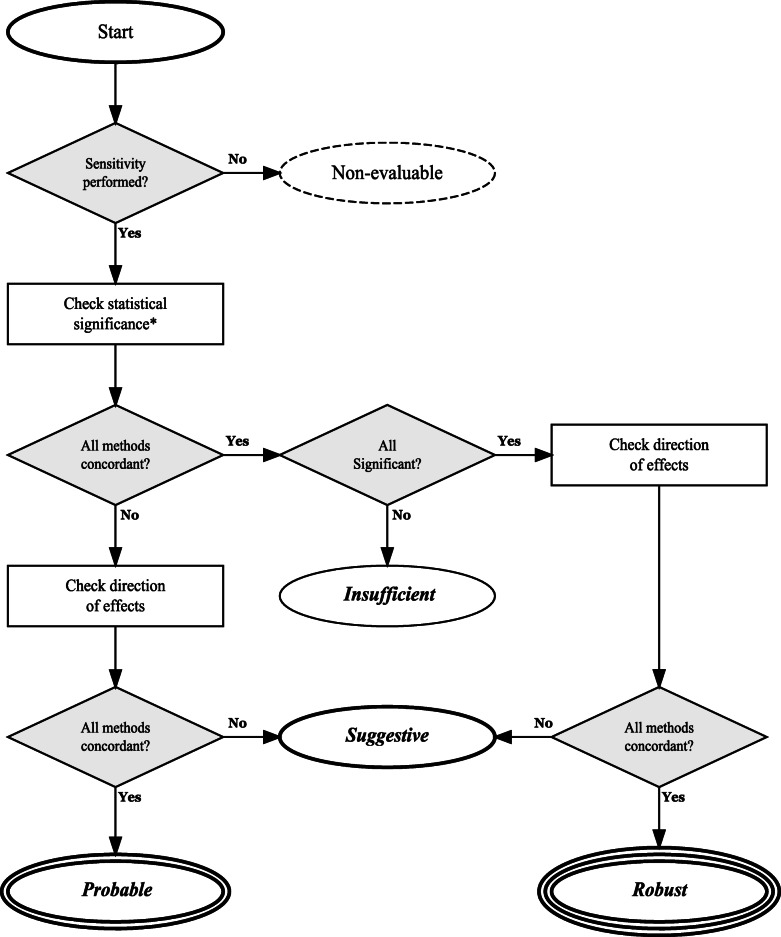
Categorization of the evidence. * For the main analysis: statistically significant at the threshold set up by the study due to multiple testing or at 0.05 if no multiple testing threshold was defined. For the sensitivity analyses: statistically significant at 0.05

The robustness of the evidence was categorized into four a priori designed levels of evidence for causality (*robust*, *probable, suggestive, insufficient* evidence) (Fig. 1) based on information from both the main MR analysis and at least one of the MR-Egger, WM, MRPRESSO, and MVMR. These methods were chosen as they are the most commonly used in the MR literature to assess and adjust for potential assumption violations. The grading was performed in the following manner: *Robust* evidence for causality was achieved when all the performed methods (i.e., main analysis, and MR-Egger, WM, MRPRESSO, and MVMR) for the specific association presented a nominally significant *p* value. We used instead the *p* value threshold for the main analysis adjusted for multiple testing when this was reported. Furthermore, in all methods, the direction of the effect estimates needed to be concordant. The evidence was graded even if some of the sensitivity analyses were not performed, but at least one was required for the evaluation. *Probable* evidence for causality was achieved when at least one method (main or sensitivity analysis) had a nominally significant *p* value of 0.05 (for the main analysis, we took the *p* value threshold as set up by the study due to multiple testing) and direction of the effect estimate was concordant for all the methods. *Suggestive* evidence for causality was achieved when at least one method had a nominally significant *p* value (for the main analysis, we took the *p* value threshold as set up by the study due to multiple testing), but the direction of the effect estimates differed between methods. Associations that presented nominally non-significant *p* value for all methods (in the main analysis, the *p* value did not survive the threshold set up by the study due to multiple testing) were classified as *insufficient* evidence for causality. This category may contain associations for which evidence for causality is unclear (due to low power and wide confidence intervals) but also associations for which MR analyses suggest that a moderate size of causal effect is unlikely. Finally, associations that did not present any of the sensitivity analyses were categorized as *non-evaluable* evidence. We also performed a separate analysis by removing the MR-Egger test from the criteria as it often provides different results from the other methods due to low power [27, 28]. Associations presenting MR-Egger as the sole sensitivity analysis were not graded in this separate evaluation.

The structure of this evidence quality grading relates more to polygenic MR analyses than to MR analyses for gene products (e.g. proteins) that are conducted using variants from a *cis*-gene window and are more likely to use only one or a few SNPs as instrument. Therefore, we further assessed the associations in the *non-evaluable* evidence category by evaluating how many of them used biological relevance and *cis* IV definitions and among them how many conducted a colocalization analysis, which evaluates the shared, local genetic architecture and causality between two traits [29].

## Patient and public involvement

No patients were involved in the development of the research question or the outcome measures, nor were they involved in the study design or the interpretation of the results.

## Results

**Fig. 2:**
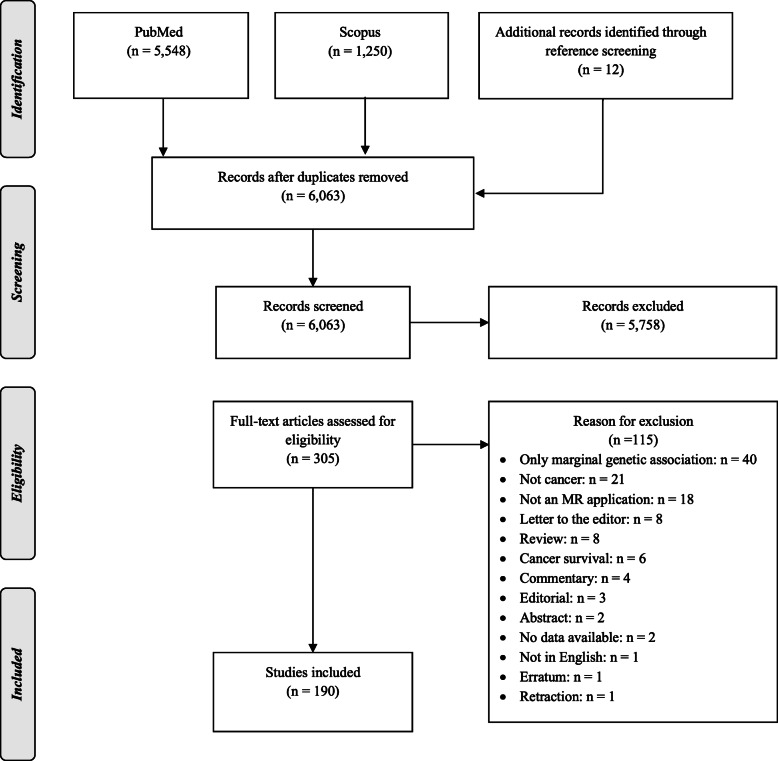
Study selection flowchart

The search strategy yielded a total of 6074 original search results of which 305 were evaluated in full text and 115 records were excluded [12, 14, 15, 20–22, 30–138] (specific reasons for exclusion are presented in Additional file 2: File S1) leading to 190 eligible MR publications [139–328] (Fig. 2). These 190 publications presented 4667 MR associations for 16 exposure categories, including 852 unique exposures, namely amino acids and derivatives (*N* = 81 unique exposures), anthropometrics (*N* = 47), circulating leukocyte telomere length (*N* = 1), diabetes and related biomarkers (*N* = 37), dietary intake and micronutrient concentrations (*N* = 42), fatty acids and derivatives (*N* = 59), growth factors (*N* = 12), inflammatory biomarkers (*N* = 82), lifestyle, education and behavior (*N* = 35), lipid metabolism biomarkers (*N* = 148), methylations (*N* = 14), reproductive factors (*N* = 8), steroids (*N* = 24), clinical measurements (*N* = 21), other diseases and traits (*N* = 47), and other metabolites/biomarkers (*N* = 194) (Additional file 2: File S2), and 21 cancer sites (i.e. head and neck, esophageal, stomach, small intestine, colorectal, liver and biliary tract, pancreatic, lung, skin/melanoma, sarcomas, breast, cervical, endometrial, ovarian, prostate, kidney, bladder and urinary tract, central nervous system, thyroid, leukemias and lymphomas, and any cancer/mixed) and their subsites. The vast majority of associations (*N* = 4532; 97%) investigated cancer risk with only 135 (3%) associations being on cancer mortality. The complete evidence base of the extracted information is provided in the Additional file 2: File S3.

### Description of the evidence base

**Fig. 3:**
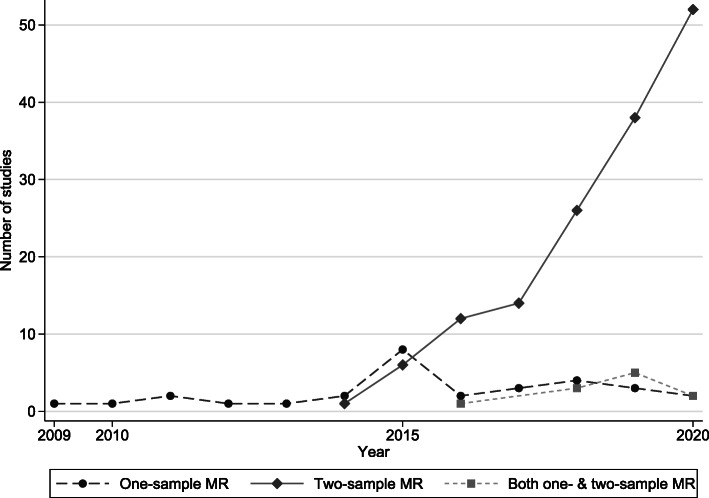
Time trend of Mendelian randomization (MR) publications on cancer risk or mortality, by MR design

The 190 MR studies on cancer were published as early as 2009, but the majority (*N* = 135; 71%) were published after 2018. Most publications (*N* = 149; 78%) used a two-sample MR design, 30 publications (15.7%) used a one-sample design, and 11 publications (5.8%) presented both one- and two-sample MR analyses. The design of one publication was unclear (Fig. 3).

For most MR analyses, the variants used as instruments for the exposure were derived from populations of European ancestry (*N* = 3183; 68.2%), 31 (0.7%) from Asian, four (0.1%) Amish, three (0.1%) South American, and 56 (1.2%) mixed, while for 1390 (29.8%) associations, the exposure population ancestry was not reported. Regarding the outcome, in most comparisons (3221; 69%) population ancestry was European, 233 (5%) Asian, 12 (0.3%) South American, one African, and 101 (2.2%) mixed, while for 1099 (23.5%) outcome population ancestry was not reported.

Body mass index (BMI) was the most frequently studied exposure with 278 MR analyses across 40 publications, followed by vitamin D-related phenotypes with 149 MR analyses across 25 publications, and height with 109 MR analyses across 23 publications. The sample size for the exposure genetic analysis was reported in 3454 associations with a median of 17,649 participants (range, 231 for the metabolite *X-12435* to 1232091 for smoking initiation).

The most frequently studied cancer was breast, which was investigated in 63 publications, followed by lung (*N* = 57), colorectal (*N* = 53), and prostate (*N* = 49). In contrast, pancreatic cancer had the highest number of MR analyses (*N* = 646; 13.8%), followed by lung (*N* = 634; 13.6%), breast (*N* = 586; 12.6%), and ovarian (*N* = 582; 2.5%). With regards to the number of cases, breast cancer had the highest number of cases (median *N* = 69,501 across 534 analyses), followed by prostate cancer (median *N* = 44,825 across 352 analyses), with small intestine cancer having the smallest median number of participants (*N* = 156; 36 analyses).

### Description of the instrument selection

The median number of SNPs used as instruments was five, ranging from one to 3163, whereas for 141 (3%) MR analyses this information was not reported (Additional file 2: Table S1). In the majority of the analyses (4108; 88%), instrument selection was based on the genome-wide significance threshold 5 × 10^−8^, 87 (1.9%) analyses used a stricter threshold of significance, 102 (2.2%) analyses used a more lenient threshold, and in 370 (7.9%) analyses the significance threshold for instrument selection was not reported. For 1241 (26.6%) associations, the authors reported that the choice of the instruments was based on their biological relevance to the exposure of interest. The most frequently used clumping thresholds for SNP inclusion were *r*^2^ < 0.001 (*N* = 1203; 25.9%), *r*^2^ < 0.01 (*N* = 1058; 22.7%), and *r*^2^ < 0.1 (*N* = 1059; 22%). The percentage of variance explained (*R*^2^) was reported for 2162 (46.3%) associations and ranged from 0.01 to 100% (for chemokine [C-X-C motif] ligand 1 and chemokine [C-C motif] ligand 4) with a median of 2.9% (Additional file 2: Table S1). Only about one-in-four associations (*N* = 1135) reported a numerical estimation of the power of the MR analysis, with a median reported power of 76% (range 1 to 100%) (Additional file 2: Table S1). A total of 1326 (28%) associations reported on the adjustments used for the exposure GWAS. The majority (*N* = 1283; 96.8%) adjusted for population stratification, 907 (68.4%) adjusted for age, 720 (54.3%) for sex, and 271 (20.4%) used adjustments specific to genotyping methods. Other adjustments included study location or assessment center (*N* = 169; 12.8%), anthropometrics (*N* = 85; 6.4%), lifestyle factors (*N* = 73; 5.5%), and study year/time (*N* = 42; 3.1%), whereas in 81 (1.7%) analyses a number of additional adjustment factors were used.

### Description of the results and robustness of the evidence

Most analyses were based on a two-sample (*N* = 4304; 92.2%) and only 363 (7.8%) used a one-sample design. The statistical analysis method of preference as main analysis with 2974 (63.7%) associations was the inverse-variance weighted method (either fixed-effect or random-effects), whereas 734 (15.7%) associations were derived from likelihood-based analyses. Other statistical analysis approaches used for the main MR analysis included the Wald ratio, generalized models (generalized least squares and generalized summary-based MR), two-stage regression approaches (35% of the one-sample designs), WM, and MR using robust-adjusted profile scores. Forty-two publications (22.1%) performed an adjustment for multiple comparisons, and from the 4667 total associations only 523 (11.2%) were statistically significant in the main analysis at the threshold set up by the study due to multiple testing or at nominal significance (*p* value < 0.05) if no multiple testing threshold was defined. Sensitivity analyses were mostly performed in two-sample MR, and a limited number of these sensitivity analyses were performed in one-sample MR designs.

Across two-sample designs, MR-Egger was evaluated in 1293 (30%) analyses with 140 (10.8%) of those presenting a nominally statistically significant MR-Egger slope; a total of 1055 (24.5%) associations performed a WM analysis with 217 (20.6%) being statistically significant, while sensitivity analyses using MRPRESSO or multivariable MR were fairly limited with only 142 (3.3%; with *N* = 55; 38.7% statistically significant) and 171 (4%; with *N* = 53; 31% statistically significant) associations, respectively (Additional file 2: Table S2). Across the 363 analyses with one-sample design, 46 performed a MR-Egger (*N* = 3; 6.5% significant), 27 a WM (*N* = 5; 18.5% significant), no analysis performed MRPRESSO, and 27 performed a MVMR analysis (*N* = 9; 33.3% significant) (Additional file 2: Table S2).

A total of 1467 (31.4%) MR associations reported in 121 publications presented results on both the main and at least one sensitivity analysis and were further evaluated based on the aforementioned grading scheme. The rest of the MR associations (*N* = 3200; 68.6%) across 123 publications only presented results for the main analysis and therefore could not be graded. Of those 3200 associations, 293 (9.2%) had a one-sample and 2907 (90,8%) a two-sample design. For 36.6% (*N* = 1171) of analyses, the authors selected the IVs based on their biological relevance to the exposure, with 1106 (94.5%) of them having a two-sample design. A total of 238 (7.4%) associations with only a main analysis were statistically significant (or survived a multiple testing threshold) and for only 60 (25.2%) of those the selection of the instrument was based on biological relevance. Of those, 14 used a *cis* definition for the selected instruments, but none of those performed a colocalization analysis.

**Fig. 4:**
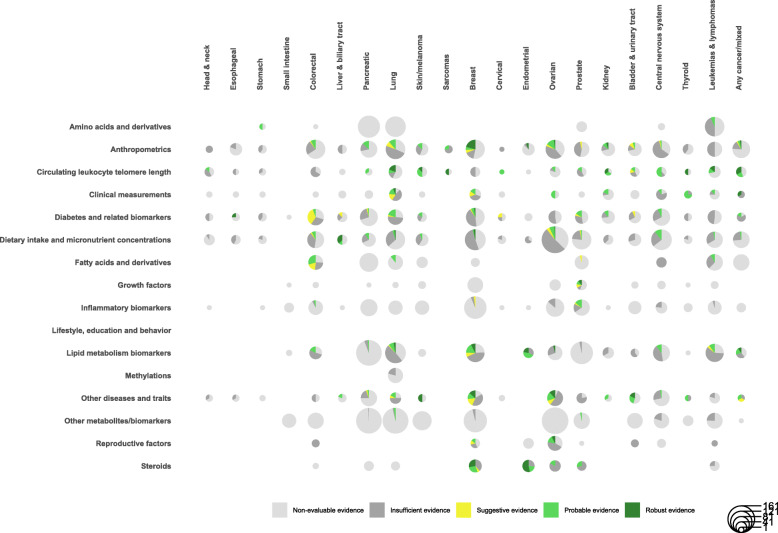
Evidence map

**Fig. 5:**
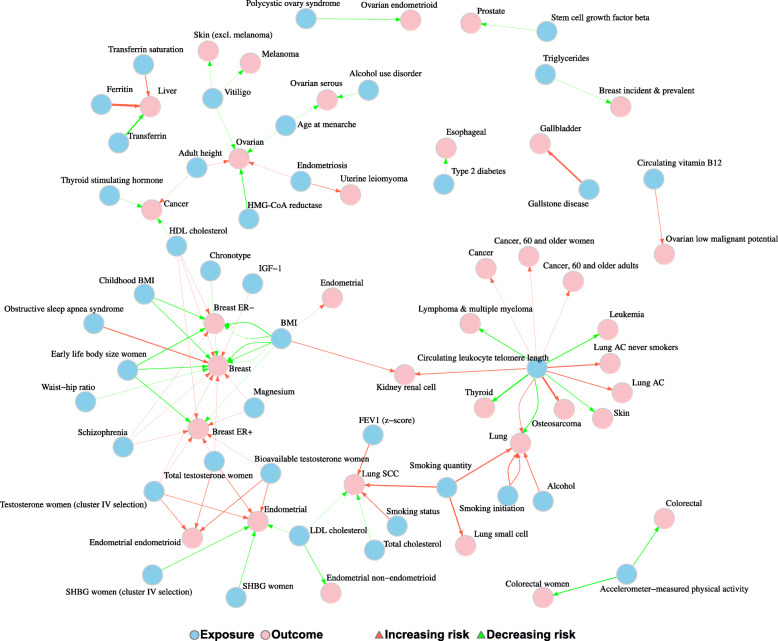
Network of the exposure–cancer associations of the Mendelian randomization analyses presenting robust evidence. Note: For circulating telomere length, the red arrows refer to longer while the green arrows refer to shorter genetically predicted telomere length. For HMG-GoA reductase, the green arrow to ovarian cancer refers to decreased genetically predicted levels of the exposure. Abbreviations: AC: adenocarcinoma; BMI: body mass index; ER−: estrogen receptor negative; ER+: estrogen receptor positive; FEV1: forced expiratory volume in one second; HDL: high-density lipoprotein; HMG-CoA: 3-Hydroxy-3-methylglutaryl coenzyme A; IGF-1: insulin-like growth factor 1; LDL: low-density lipoprotein; SCC: squamous cell carcinoma; SHBG: sex-hormone-binding globulin

A graphical overview of the robustness of the evidence per exposure category and cancer group is presented in Fig. 4. Out of the 1467 graded associations, we observed 87 MR analyses that presented *robust* evidence (5.9%; 1.9% of total MR analyses), 275 with *probable* evidence (18.8%; 5.9% of total), 89 with *suggestive* evidence (6.1%; 1.9% of total), and 1016 with *insufficient* evidence (69.3%; 21.8% of total) based on the results of the main and sensitivity analyses. Across the 16 exposure categories, anthropometrics had the highest number of *robust* analyses (*N* = 16; 18.4%), followed by steroids (*N* = 13; 15%), circulating leukocyte telomere length (*N* = 13; 15%), the other diseases and traits category (*N* = 12; 13.8%), and lipids (*N* = 10;11.5%), whereas no *robust* association was found among the amino acids and derivatives, fatty acids and derivatives, inflammatory biomarkers, methylations, and other metabolites and biomarkers categories (Table 1). Across cancers, the highest number of *robust* associations was observed for breast cancer with 29 (33.3%) of the 87 *robust* associations, followed by lung cancer (*N* = 14; 16.1%) and endometrial (*N* = 11; 12.6%). Head and neck, stomach, small intestine, pancreatic, cervical, and central nervous system cancers did not present any *robust* MR associations (Table 2). The network of the *robust* exposure–cancer associations is presented in Fig. 5.

**Table 1:** Number and percent of Mendelian randomization analyses per grading category by exposure category

Exposure category	Robust evidence	Probable evidence	Suggestive evidence	Insufficient evidence	Non-evaluable
**Amino acids and derivatives**	0 (0)	5 (1.82)	0 (0)	27 (2.66)	210 (6.56)
**Anthropometrics**	16 (18.39)	37 (13.45)	16 (17.98)	177 (17.42)	299 (9.34)
**Circulating leukocyte telomere length**	13 (14.94)	20 (7.27)	1 (1.12)	25 (2.46)	68 (2.13)
**Clinical measurements**	2 (2.3)	14 (5.09)	5 (5.62)	25 (2.46)	53 (1.66)
**Diabetes and related biomarkers**	2 (2.3)	22 (8)	20 (22.47)	121 (11.91)	188 (5.88)
**Dietary intake and micronutrient concentrations**	7 (8.05)	31 (11.27)	8 (8.99)	235 (23.13)	371 (11.59)
**Fatty acids and derivatives**	0 (0)	14 (5.09)	6 (6.74)	27 (2.66)	187 (5.84)
**Growth factors**	1 (1.15)	1 (0.36)	1 (1.12)	1 (0.1)	72 (2.25)
**Inflammatory biomarkers**	0 (0)	6 (2.18)	3 (3.37)	22 (2.17)	347 (10.84)
**Lifestyle, education and behavior**	9 (10.34)	48 (17.45)	9 (10.11)	66 (6.5)	108 (3.38)
**Lipid metabolism biomarkers**	10 (11.49)	35 (12.73)	7 (7.87)	144 (14.17)	344 (10.75)
**Methylations**	0 (0)	0 (0)	0 (0)	6 (0.59)	23 (0.72)
**Other diseases and traits**	12 (13.79)	21 (7.64)	11 (12.36)	67 (6.59)	96 (3)
**Other metabolites/biomarkers**	0 (0)	4 (1.45)	0 (0)	21 (2.07)	783 (24.47)
**Reproductive factors**	2 (2.3)	5 (1.82)	1 (1.12)	24 (2.36)	29 (0.91)
**Steroids**	13 (14.94)	12 (4.36)	1 (1.12)	28 (2.76)	22 (0.69)
**Total**	**87 (100)**	**275 (100)**	**89 (100)**	**1016 (100)**	**3200 (100)**

**Table 2:** Number and percent of Mendelian randomization analyses per grading category by cancer group

Cancer group	Robust evidence	Probable evidence	Suggestive evidence	Insufficient evidence	Non-evaluable
**Head and neck**	0 (0)	2 (0.73)	0 (0)	10 (0.98)	23 (0.72)
**Esophageal**	1 (1.15)	1 (0.36)	0 (0)	8 (0.79)	28 (0.88)
**Stomach**	0 (0)	3 (1.09)	0 (0)	7 (0.69)	20 (0.63)
**Small intestine**	0 (0)	0 (0)	0 (0)	0 (0)	36 (1.13)
**Colorectal**	2 (2.3)	31 (11.27)	21 (23.6)	75 (7.38)	156 (4.88)
**Liver and biliary tract**	3 (3.45)	2 (0.73)	1 (1.12)	5 (0.49)	29 (0.91)
**Pancreatic**	0 (0)	15 (5.45)	2 (2.25)	42 (4.13)	587 (18.34)
**Lung**	14 (16.09)	46 (16.73)	14 (15.73)	148 (14.57)	412 (12.88)
**Skin/melanoma**	3 (3.45)	7 (2.55)	0 (0)	14 (1.38)	136 (4.25)
**Sarcomas**	1 (1.15)	1 (0.36)	0 (0)	3 (0.3)	1 (0.03)
**Breast**	29 (33.33)	40 (14.55)	20 (22.47)	140 (13.78)	357 (11.16)
**Cervical**	0 (0)	2 (0.73)	1 (1.12)	3 (0.3)	14 (0.44)
**Endometrial**	11 (12.64)	7 (2.55)	0 (0)	10 (0.98)	31 (0.97)
**Ovarian**	9 (10.34)	35 (12.73)	11 (12.36)	180 (17.72)	347 (10.84)
**Prostate**	1 (1.15)	15 (5.45)	6 (6.74)	57 (5.61)	278 (8.69)
**Kidney**	2 (2.3)	9 (3.27)	1 (1.12)	17 (1.67)	55 (1.72)
**Bladder and urinary tract**	2 (2.3)	6 (2.18)	5 (5.62)	23 (2.26)	116 (3.62)
**Central nervous system**	0 (0)	22 (8)	1 (1.12)	110 (10.83)	201 (6.28)
**Thyroid**	1 (1.15)	4 (1.45)	0 (0)	9 (0.89)	34 (1.06)
**Leukemias and lymphomas**	2 (2.3)	16 (5.82)	2 (2.25)	125 (12.3)	213 (6.66)
**Any cancer/mixed**	6 (6.9)	11 (4)	4 (4.49)	30 (2.95)	126 (3.94)
**Total**	**87 (100)**	**275 (100)**	**89 (100)**	**1016 (100)**	**3200 (100)**

The 16 *robust* associations from the anthropometrics category pertained to BMI (including childhood BMI and early life body size) and waist-to-hip ratio (WHR) with decreased risk of total breast cancer [164, 250, 255, 299], estrogen receptor positive (ER+) [250, 299], and negative (ER−) disease [164, 250, 299]); BMI with increased risk of kidney/renal cell [240] and endometrial [293] cancer, and adult height with increased overall [204] and ovarian cancer risk [194]. Thirteen *robust* associations were observed in the steroids category, pertaining to the positive association of different measures of testosterone with breast (total, ER+) and endometrial cancer, and to the negative association of sex-hormone-binding globulin (SHBG) and endometrial cancer [301]. Thirteen *robust* associations were also found for longer (shorter) leukocyte telomere length pertaining to increased (decreased risk, respectively) risk of total cancer [244], lung (total, adenocarcinoma [AC], AC-never smokers) [241], kidney/renal cell [185], osteosarcoma [314], skin [288], thyroid [288], leukemia [288], and lymphoma and multiple myeloma [288]. The 10 *robust* associations from the lipid metabolism biomarkers category pertained to high-density lipoprotein cholesterol (HDL-C) with increased risk of breast (total, ER+, ER−) [279] but decreased risk of overall cancer [197]; triglycerides (TGL) with decreased risk of breast [207]; low-density lipoprotein cholesterol (LDL-C) with decreased risk of endometrial (total, non-endometrioid) [321] and lung squamous cell carcinoma (SCC) [178]; total cholesterol and lung SCC (decreased risk) [178]; and 3-hydroxy-3-methylglutaryl coenzyme A (HMG-CoA) reductase with ovarian cancer (decreased risk for decreased genetically predicted levels of the exposure) [309]. From the lifestyle, education, and behavior category, six associations were found with robust evidence, namely between smoking and increased risk of lung cancer (total [286, 328], SCC [328], small cell [328]), two between physical activity and decreased risk of colorectal cancer [296] and one between chronotype and decreased risk of breast cancer [254]. From the dietary intake and micronutrient concentrations category, we found eight *robust* associations pertaining to magnesium with breast (total and ER+, increased risk) [324], ferritin with liver (increased risk) [311], alcohol consumption with lung (increased risk) [286], and vitamin B12 with increased risk of ovarian cancer of low malignant potential [274]. Transferrin saturation showed increased risk of liver cancer, but transferrin levels presented a decreased risk [311]. The rest of the *robust* associations pertained to age at menarche with ovarian (total and serous; decreased risk) [260], alcohol use disorder diagnostic codes with ovarian serous (decreased risk) [317], endometriosis with ovarian [261] and with endometriosis-uterine leiomyoma [235] (both increased risk), gallstone disease with gallbladder (increased risk) [264], insulin-like growth factor 1 (IGF-1) with breast (increased risk) [295], obstructive sleep apnea syndrome with breast (increased risk) [271], polycystic ovary syndrome with ovarian endometrioid (decreased risk) [237], stem cell growth factor beta (SCGF-β) with prostate (decreased risk) [304], schizophrenia with breast (total, ER+, ER−; increased risk) [210], standardized forced expiratory volume in 1 s with lung SCC (increased risk) [281], thyroid-stimulating hormone with cancer overall (decreased risk) [313], type 2 diabetes with esophageal (decreased risk) [312], and vitiligo with non-melanoma skin, melanoma, and ovarian (decreased risk) [306].

When the MR-Egger test was removed from the grading scheme as a sensitivity analysis, a total of 70 associations with *probable* and four with *suggestive* evidence were upgraded to *robust*, while 35 associations were upgraded from *suggestive* to *probable*. In contrast, 23 MR analyses with *probable* and 32 with *suggestive* evidence were downgraded to *insufficient* evidence. Finally, 15 associations with *robust* evidence, 34 with *probable*, 17 with *suggestive,* and 242 with *insufficient* evidence now presented only a main analysis and were *non-evaluable* (Additional file 2: Table S3).

## Discussion

In this large systematic overview, we searched and mapped current literature evaluating the association of 852 distinct genetically predicted risk factors across 16 broad exposure categories in relation to 21 cancer sites and their subtypes by evaluating the results of 190 publications and over 4600 MR associations. Using a set of clear, comprehensive and easily replicable criteria to evaluate the validity of the reported associations, we found that less than 90 of the reported MR analyses presented *robust* evidence for causality and that the vast majority of the analyses did not perform sensitivity analyses, at least with regard to MR-Egger, WM, MRPRESSO, and MVMR. Most of the MR analyses supported by *robust* evidence were observed for anthropometric indices, steroid hormones, telomere length, and lipids.

The median number of IV size across all analyses was relatively small (*N* = 5), despite most studies being conducted in an era of large GWASs across a wide breadth of phenotypes. This may partially be explained by the large number of infrequently used biomarkers that were assessed in some studies [245, 315]. This may have affected the implementation of sensitivity analyses such as MR-Egger in several cases that did not include enough IVs. However, in only a limited number of analyses a further exploration of the association was performed using other approaches such as colocalization. Apart from sensitivity MR analyses not being frequently performed in the original studies (often but not always due to lack of sufficient number of IVs), other valuable insights regarding the methodological approaches can be gained by examining this evidence base. We observed that several different clumping thresholds for pruning SNPs were applied. While most studies used thresholds ranging from *r*^2^ < 0.001 to *r*^2^ < 0.1, one in ten had an even more liberal threshold. Researchers should consider adjusting for the potential correlation between IVs when using less strict thresholds such as 0.1 or higher [329]. Of note is also that less half of the analyses provided the percentage of variance explained by the IV and less than one quarter provided a power estimation, although some studies presented the power estimations graphically, but we were not able to extract those. Both the *R*^2^ and a priori power estimation are equally important for evaluating the capacity of an IV to provide valid and accurate estimates and can help to differentiate between non-significant but otherwise underpowered associations from real null ones.

Across the MR analyses pertaining to anthropometric exposures, *robust* evidence was observed predominantly for BMI. BMI was inversely associated with risk of total, ER+, and ER− breast cancer (mostly post-menopausal), which was supported by *robust* evidence across several different MR analyses. In contrast, observational evidence supports a positive association of body fatness with post-menopausal breast cancer risk, and an inverse association for premenopausal disease [22, 330, 331]. These contradictory results between MR and observational evidence may be attributed to the fact that genetically predicted BMI reflects more closely early life body fatness [164, 332], and early life body fatness has been inversely associated in observational [333] and in MR studies [164, 299] with both pre- and post-menopausal breast cancer. *Robust* evidence was also observed for a positive association of BMI and endometrial cancer in Asian populations [293], which is in line with the observational evidence on body fatness and endometrial cancer in the general population [330, 334, 335]. The results were also consistent in the main analysis of the four MR publications on BMI and endometrial cancer among European populations; however, these publications did not perform any sensitivity analyses for endometrial cancer [149, 203, 236], so they could not be evaluated in our grading scheme. The positive association of body fatness with renal cell carcinoma from observational studies [330, 336, 337] was confirmed in our review based on *robust* evidence for BMI and *probable* evidence for WHR and body fat percentage, both of which were upgraded to *robust* in the sensitivity analysis excluding the MR-Egger analysis. Several well-acknowledged observational associations of adiposity and cancer risk, namely for ovarian [330, 334, 338] and colorectal [330, 339] cancer were only supported by *probable* evidence. The association for ovarian cancer from the largest MR study to-date failed to reach *robust* evidence due to the main analysis not surviving the multiple comparisons threshold set by the original publication that investigated many risk factors, despite being nominally significant [261]. Similarly, for colorectal cancer, the MR analyses, despite consistently indicating an increased risk [164, 167], did not reach *robust* evidence due to several reasons, including not surviving the multiple correction thresholds and having non-significant sensitivity analyses. BMI also presented *probable* evidence of an increased risk with lung SCC. The results from observational data are showing inverse associations for BMI and risk of total lung cancer [330, 340], which are likely due to residual confounding by smoking [341]. With respect to other anthropometric exposures, namely adult height, WHR, waist and hip circumference, the results were in line with the ones for BMI although being supported by lower levels of evidence in MR studies, with the exception of adult height and overall [204] and ovarian cancer [194] that reached *robust* evidence.

*Robust* and *probable* evidence was also found for the positive association of genetically predicted testosterone concentrations with risk of breast and endometrial cancer, and the negative association of SHBG with endometrial cancer. These results have been partially confirmed in observational evidence [342, 343]. Conversion of androgens into estrogens in the adipose tissue of post-menopausal women may partially explain these results, due to the role of estrogens in breast [344] and endometrial cancer cell proliferation [345]. On the other hand, excess weight, insulin resistance, and hyperinsulinemia have been associated with changes in total and bioavailable plasma sex steroid levels in women through a number of mechanisms that can lead to a decrease in plasma SHBG levels, and a rise in bioavailable testosterone [346].

A considerable fraction of the studies focused on circulating leukocyte telomere length, for which *robust* associations were observed with total cancer, and with lung, leukemia, lymphoma, osteosarcoma, skin, and thyroid cancers, where longer telomeres increased the risk (or shorter lengths decreased the risk) of these cancers. Furthermore, a positive association with increased telomere length was supported by *probable* evidence for a number of other cancer sites, such as glioma, bladder, kidney, melanoma, multiple myeloma, non-Hodgkin’s lymphoma, ovarian, and prostate cancer, several of which were upgraded to *robust* with the exclusion of the MR-Egger analysis. In contrast, negative associations of increased telomere length with cervical, head and neck, pancreatic, and skin basal cell cancers were supported by *probable* evidence. The observational evidence has created controversy in the literature about the direction of the associations [347, 348], while in a recent umbrella review the strength of the observational evidence was deemed relatively weak and inconsistent [349]. A recent review on the association of telomere length and cancer risk highlighted the importance of the pleiotropic effects of certain telomere-related loci such as *TERT*, *TERC*, and *OBFC1* [20], while mediation MR analyses have indicated that a considerable proportion of the association between the *TERT* region and lung cancer risk is mediated by telomere length [241]. The current understanding is that telomeres may both promote and also limit cancer proliferation and neoplastic progression [350, 351], although the potential of proliferation from longer telomeres seemingly overshadows the risk stemming from genetically determined shorter telomeres [352].

Several associations were identified for lipids, especially TGL, total cholesterol, LDL-C, and HDL-C. Specifically, the negative association of TGL with total and ER+ breast cancer was supported by *robust* and *probable* evidence, which is in line with the observational evidence [353, 354]. For LDL-C and HDL-C, the MR results were consistent across several studies, indicating a positive association with total, ER+, and ER− breast cancer. These associations are further supported by consistent results from MVMR analyses adjusting for other lipid traits. However, the observational evidence is contradictory for LDL-C and HDL-C, as previous meta-analyses have shown a negative association for LDL-C and no association for HDL-C [354, 355]. With regard to endometrial cancer, we found *robust* evidence for a negative association with LDL-C and lower levels of evidence for associations with other lipids [321]. These results were concordant with MVMR analyses adjusting for BMI, but further MVMR analyses mutually adjusting for lipids were not performed. Limited observational evidence indicates a positive association with TGL [356–358] but no association with LDL-C or HDL-C [356, 359, 360]. An emerging *robust* association was observed between HMG-CoA reductase, the drug target of statins, and lower risk of ovarian cancer with consistent MVMR results accounting for BMI. Observational evidence for statin use suggests a decreased risk of ovarian cancer among statin users [361]. Only two associations presented *robust* evidence with lung SCC, pertaining to a negative association for total cholesterol and LDL-C, but MVMR analyses were not conducted, while for total lung cancer these associations were supported by *probable* evidence. Observational studies indicated a lower risk of lung cancer for circulating lipids [362]. For several other cancers such as colorectal, glioma, lymphomas, pancreatic, kidney, and multiple myeloma, the MR results were limited and inconsistent, without any *robust* evidence. The role of lipid metabolism in carcinogenesis and tumor growth has been acknowledged in the literature [363, 364] although the molecular mechanism is not yet fully understood and the associations are complicated by the potential role of different lipid subfractions and correlation between different lipids as well as with other traits and diseases such as BMI or metabolic syndrome [365, 366]. Regulating lipid metabolism has been identified as a promising target for anti-cancer interventions [363]. An overview of reviews on statin use has shown low levels of evidence in meta-analyses of observational studies for decreased risk of breast, colorectal, esophageal, gastric, hematological, liver, and prostate cancers, while the results from meta-analyses of RCTs were null [367].

Many of the included associations were non-evaluable due to not performing any of the sensitivity analyses required for our grading. Reasons may vary across studies, including inability to do so due to low number of instruments, especially for the MR-Egger analyses, prioritization of statistically significant associations for further evaluation with sensitivity analyses, or sensitivity analyses not being part of the authors’ analysis plan. There is a necessity to study these associations more comprehensively, especially in the cases of polygenic definition of instruments, which are more prone to biases or pleiotropy that can drive associations both towards and away from the null. Regardless of the reason and the appropriateness of the decision to include sensitivity MR analyses, these associations are not sufficiently investigated and are all considered non-evaluable in our grading scheme, which focuses on evaluating the robustness for causality of the studied associations.

Other efforts to summarize the evidence of MR analyses on cancer risk have been performed previously. However, they were either limited to specific exposures [12, 14, 18, 20] or cancer sites [15, 16], or used a more narrative approach of presenting and assessing the MR results [11, 13, 19], while none performed a formal evaluation of the evidence. Instead, our review used predefined criteria for the categorization of the evidence for causality, which increases the transparency and reproducibility of our results. We did not evaluate the quality of reporting of the MR studies, as there are only some very recent efforts focusing in this topic [17], and comprehensive reporting guidelines were very recently developed [7]. In addition, as guidelines for performing MR studies [6] have also very recently been developed and are not yet widely agreed upon, we refrained from using those to evaluate the quality of the identified studies. Although the grading scheme utilized in our review prohibited us from evaluating a large proportion of the included MR analyses because they did not report on any sensitivity MR analysis, most of the results that received robust evidence were in line with previous observational research and are further supported by mechanistic evidence.

Several limitations need to be acknowledged. Our search strategy may have resulted in missing some relevant studies, especially if the MR analysis was not the primary focus of some studies but only a supplementary analysis, which seems to be increasingly common in recent GWA studies. In these cases, however, we would not expect a comprehensive evaluation of the studied associations using sensitivity MR analyses, which would only lead to inflation of the number of associations with *non-evaluable* evidence. The structure of the criteria for evaluation of the robustness of the MR evidence for causality was more geared towards the evaluation of two-sample MR approaches, but the percentage of one-sample designs that did not perform one of the pre-specified sensitivity analyses was only marginally higher than that of two-sample designs. Associations evaluated in earlier publications, especially those before many of the sensitivity analyses were introduced, could also not be evaluated. However, the majority of the studies were published after 2018 and the earlier associations often relied on limited number of cases or on instruments including only a limited number of SNPs and with low percentage of variability explained. Information of the percentage of variance explained and statistical power of the instrument was often not reported, and thus a complete assessment of weak instrument bias could not be performed. Therefore, the grading scheme did not allow us to distinguish MR analyses that presented robust evidence of lack of association from MR analyses that did not present an association due to being insufficiently powered. Future studies may benefit from reporting this information. The approach undertaken in this review for grading the associations did not allow to us to evaluate MR analyses that only presented a main analysis without being supported by sensitivity analyses. Since two of the three MR assumptions are not directly testable, a MR analysis is imperative to be supported by a comprehensive evaluation of complementary and sensitivity analyses to increase credibility of the results, as such approaches can at least give some indication of large violations of the assumptions. Most MR analyses evaluating associations for gene products using *cis* instruments were non-evaluable using our current criteria as most included one or two SNPs as IVs, and the sensitivity analyses could not be applied. However, only two of these studies performed colocalization analysis and neither presented statistically significant associations for these specific analyses. More recently introduced sensitivity MR analyses were not included in the current evaluation, as their use is very infrequent in the MR literature. Finally, there is discrepancy in the availability of genetic data for different cancers, and hence the MR studies that have been possible; thus, cancer consortia are encouraged to make their summary data more readily and widely available.

## Conclusions

The field of cancer epidemiology is challenging to evaluate due to the sheer amount of available observational evidence and further burdened by the increasing interest on MR methodologies that could complement findings from traditional observational research. Our work summarizes and evaluates the robustness of the MR analyses evidence for causality in cancer prevention and etiology. Only a minority of the evaluated MR analyses were supported by *robust* evidence. In addition, we identified gaps in the conduct and reporting of MR studies that will assist in developing stronger future reporting guidelines.

## Supplementary Information

**Additional file 1.** Supplementary methods.

**Additional file 2: File S1.** List of excluded studies and reason for exclusion. **File S2.** List of distinct exposures per exposure category. **File S3.** Evidence base. **Table S1.** Description of instrument characteristics by exposure category. **Table S2.** Summary results of the main and sensitivity Mendelian randomization (MR) analyses by exposure category and cancer group. **Table S3.** Grading of the evidence based on the sensitivity analysis by excluding the results from the MR-Egger test.

## Data Availability

The data underlying this article are available in the supplement and in the original publications.

## Abbreviations

AC: Adenocarcinoma
BMI: Body mass index
ER-: Estrogen receptor negative
ER +: Estrogen receptor positive
FEV1: Forced expiratory volume in one second
GWAS: Genome-wide association studies
HDL-C: High-density lipoprotein cholesterol
HMG-CoA: 3-Hydroxy-3-methylglutaryl coenzyme A
IGF-1: Isulin-like growth factor 1
IV: Instrumental variable
LDL-C: Low-density lipoprotein cholesterol
MR: Mendelian randomization
MVMR: Multivariable Mendelian randomization
PRISMA: Preferred Reporting Items for Systematic Review and Meta-Analysis
RCT: Randomized controlled trial
SCC: Squamous cell carcinoma
SCGF-β: Stem cell growth factor beta
SHBG: Sex-hormone-binding globulin
SNPs: Single-nucleotide polymorphism
TGL: Triglycerides
WHR: Waist-to-hip ratio
WM: Weighted median

## References

[1] Sung H, Ferlay J, Siegel RL, Laversanne M, Soerjomataram I, Jemal A, Bray F (2021). Global cancer statistics 2020: GLOBOCAN estimates of incidence and mortality worldwide for 36 cancers in 185 countries. CA Cancer J Clin.

[2] Dwan K, Gamble C, Williamson PR, Kirkham JJ, Reporting Bias G (2013). Systematic review of the empirical evidence of study publication bias and outcome reporting bias - an updated review. PloS one.

[3] Hingorani A, Humphries S (2005). Nature’s randomised trials. Lancet (London, England).

[4] Lawlor DA, Harbord RM, Sterne JA, Timpson N, Davey Smith G (2008). Mendelian randomization: using genes as instruments for making causal inferences in epidemiology. Stat Med.

[5] Pierce BL, Burgess S (2013). Efficient design for Mendelian randomization studies: subsample and 2-sample instrumental variable estimators. Am J Epidemiol.

[6] Burgess S, Davey Smith G, Davies NM, Dudbridge F, Gill D, Glymour MM, Hartwig FP, Holmes MV, Minelli C, Relton CL (2019). Guidelines for performing Mendelian randomization investigations. Wellcome Open Research.

[7] Skrivankova VW, Richmond RC, Woolf BAR, Yarmolinsky J, Davies NM, Swanson SA, VanderWeele TJ, Higgins JPT, Timpson NJ, Dimou N (2021). Strengthening the reporting of observational studies in epidemiology using Mendelian randomization: the STROBE-MR Statement. Jama.

[8] Burgess S, Bowden J, Fall T, Ingelsson E, Thompson SG (2017). Sensitivity analyses for robust causal inference from Mendelian randomization analyses with multiple genetic variants. Epidemiology.

[9] Moher D, Liberati A, Tetzlaff J, Altman DG, Group P (2009). Preferred reporting items for systematic reviews and meta-analyses: the PRISMA statement. BMJ (Clin Res ed).

[10] Boef AG, Dekkers OM, le Cessie S (2015). Mendelian randomization studies: a review of the approaches used and the quality of reporting. Int J Epidemiol.

[11] Yarmolinsky J, Wade KH, Richmond RC, Langdon RJ, Bull CJ, Tilling KM, Relton CL, Lewis SJ, Davey Smith G, Martin RM (2018). Causal inference in cancer epidemiology: what is the role of mendelian randomization?. Cancer Epidemiol Biomark Prev.

[12] Murphy N, Jenab M, Gunter MJ (2018). Adiposity and gastrointestinal cancers: epidemiology, mechanisms and future directions. Nat Rev Gastroenterol Hepatol.

[13] Pierce BL, Kraft P, Zhang C (2018). Mendelian randomization studies of cancer risk: a literature review. Curr Epidemiol Rep.

[14] Cornelis MC, Munafo MR. Mendelian randomization studies of coffee and caffeine consumption. Nutrients. 2018;10:–10. 10.3390/nu10101343 PMC6213346 30241358

[15] Cornish AJ, Tomlinson IPM, Houlston RS (2019). Mendelian randomisation: a powerful and inexpensive method for identifying and excluding non-genetic risk factors for colorectal cancer. Mol Asp Med.

[16] O'Mara TA, Glubb DM, Kho PF, Thompson DJ, Spurdle AB (2019). Genome-wide association studies of endometrial cancer: latest developments and future directions. Cancer Epidemiol Biomark Prev.

[17] Lor GCY, Risch HA, Fung WT, Au Yeung SL, Wong IOL, Zheng W, Pang H (2019). Reporting and guidelines for mendelian randomization analysis: a systematic review of oncological studies. Cancer Epidemiol.

[18] Grant WB (2020). Review of recent advances in understanding the role of vitamin D in reducing cancer risk: breast, colorectal, prostate, and overall cancer. Anticancer Res.

[19] Gala H, Tomlinson I (2020). The use of Mendelian randomisation to identify causal cancer risk factors: promise and limitations. J Pathol.

[20] Nelson CP, Codd V (2020). Genetic determinants of telomere length and cancer risk. Curr Opin Genet Dev.

[21] Renehan AG, Zwahlen M, Egger M (2015). Adiposity and cancer risk: new mechanistic insights from epidemiology. Nat Rev Cancer.

[22] Clarke MA, Joshu CE (2017). Early life exposures and adult cancer risk. Epidemiol Rev.

[23] Bowden J, Davey Smith G, Burgess S (2015). Mendelian randomization with invalid instruments: effect estimation and bias detection through Egger regression. Int J Epidemiol.

[24] Bowden J, Davey Smith G, Haycock PC, Burgess S (2016). Consistent estimation in Mendelian randomization with some invalid instruments using a weighted median estimator. Genet Epidemiol.

[25] Verbanck M, Chen CY, Neale B, Do R (2018). Detection of widespread horizontal pleiotropy in causal relationships inferred from Mendelian randomization between complex traits and diseases. Nat Genet.

[26] Burgess S, Thompson SG (2015). Multivariable Mendelian randomization: the use of pleiotropic genetic variants to estimate causal effects. Am J Epidemiol.

[27] Minelli C, Fabiola Del Greco M, van der Plaat DA, Bowden J, Sheehan NA, Thompson J. The use of two-sample methods for Mendelian randomization analyses on single large datasets. bioRxiv. 2020:2020.2005.2007.082206. 10.1093/ije/dyab084 PMC8580269 33899104

[28] Slob EAW, Burgess S (2020). A comparison of robust Mendelian randomization methods using summary data. Genet Epidemiol.

[29] Kanduri C, Bock C, Gundersen S, Hovig E, Sandve GK (2019). Colocalization analyses of genomic elements: approaches, recommendations and challenges. Bioinformatics.

[30] Zhou H, Zhang Y, Liu J, Yang Y, Fang W, Hong S, Chen G, Zhao S, Zhang Z, Shen J (2019). Education and lung cancer: a Mendelian randomisation study. Ann Oncol.

[31] Liu J, Zhou H, Zhang Y, Fang W, Yang Y, Hong S, Chen G, Zhao S, Shen J, Xian W (2019). A Mendelian randomization study of the effects of Crohn’s disease on lung cancer. Ann Oncol.

[32] Bonilla C, Lewis SJ, Martin RM, Donovan JL, Hamdy FC, Neal DE, Eeles R, Easton D, Kote-Jarai Z, Al Olama AA (2016). Pubertal development and prostate cancer risk: Mendelian randomization study in a population-based cohort. BMC Med.

[33] Bonilla C, Lewis SJ, Rowlands MA, Gaunt TR, Davey Smith G, Gunnell D, Palmer T, Donovan JL, Hamdy FC, Neal DE (2016). Assessing the role of insulin-like growth factors and binding proteins in prostate cancer using Mendelian randomization: genetic variants as instruments for circulating levels. Int J Cancer.

[34] Guo Q, Burgess S, Turman C, Bolla MK, Wang Q, Lush M, Abraham J, Aittomäki K, Andrulis IL, Apicella C (2017). Body mass index and breast cancer survival: a Mendelian randomization analysis. Int J Epidemiol.

[35] Chou WC, Hsiung CN, Chen WT, Tseng LM, Wang HC, Chu HW, Hou MF, Yu JC, Shen CY (2020). A functional variant near XCL1 gene improves breast cancer survival via promoting cancer immunity. Int J Cancer.

[36] Xu J, Chang WS, Tsai CW, Bau DT, Xu Y, Davis JW, Thompson TC, Logothetis CJ, Gu J (2020). Leukocyte telomere length is associated with aggressive prostate cancer in localized prostate cancer patients. EBioMedicine.

[37] Langdon R, Richmond R, Elliott HR, Dudding T, Kazmi N, Penfold C, Ingarfield K, Ho K, Bretherick A, Haley C (2020). Identifying epigenetic biomarkers of established prognostic factors and survival in a clinical cohort of individuals with oropharyngeal cancer. Clin Epigenetics.

[38] Bradley D (2020). Obesity, thyroid nodularity, and thyroid cancer: epiphenomenon or cause?. J Clin Endocrinol Metab.

[39] Karantanos T, Kaizer H, Chaturvedi S, Resar LMS, Moliterno AR (2020). Inflammation exerts a nonrandom risk in the acquisition and progression of the MPN: insights from a Mendelian randomization study. EClinicalMedicine.

[40] Salaspuro M, Lachenmeier DW (2020). Unique human cancer model for acetaldehyde based on Mendelian randomization. Arch Toxicol.

[41] Bell KJL (2020). Causal inference in melanoma epidemiology using Mendelian randomization. Br J Dermatol.

[42] Boffetta P (2010). Exploring a cancer biomarker: the example of C-reactive protein. J Natl Cancer Inst.

[43] Abrams JA, Chak A (2014). Applying big GWAS data to clarify the role of obesity in Barrett's esophagus and esophageal adenocarcinoma. J Natl Cancer Inst.

[44] Schooling C M. Childhood adiposity, adult body mass index, and disease in later life. BMJ. 2020;369:m1708. 10.1136/bmj.m1708.

[45] Boccia S, Hashibe M, Galli P (2009). Aldehyde dehydrogenase 2 and head and neck cancer: A meta-analysis implementing a Mendelian randomization approach. Cancer Epidemiology Biomarkers and Prevention.

[46] Went M, Sud A, Law PJ, Johnson DC, Weinhold N, Försti A, van Duin M, Mitchell JS, Chen B, Kuiper R (2017). Assessing the effect of obesity-related traits on multiple myeloma using a Mendelian randomisation approach. Blood cancer journal.

[47] Said MA, Eppinga RN, Hagemeijer Y, Verweij N, van der Harst P (2017). Telomere length and risk of cardiovascular disease and cancer. J Am Coll Cardiol.

[48] Kar SP, Brenner H, Giles GG, Huo D, Milne RL, Rennert G, Simard J, Zheng W, Burgess S, Pharoah PDP (2019). Body mass index and the association between low-density lipoprotein cholesterol as predicted by HMGCR genetic variants and breast cancer risk. Int J Epidemiol.

[49] Zhou H, Liu J, Zhang Y, Huang Y, Zhang L (2019). Autoimmune diseases and lung cancer: a Mendelian randomization study. J Thoracic Oncol.

[50] Zhou H, Shen J, Fang W, Liu J, Zhang Y, Huang Y, Zhang L (2020). Mendelian randomization study showed no causality between metformin use and lung cancer risk. Int J Epidemiol.

[51] Barahona Ponce C, Scherer D, Boekstegers F, Garate-Calderon V, Jenab M, Aleksandrova K, Katzke V, Weiderpass E, Bonet C, Moradi T (2020). Arsenic and gallbladder cancer risk: Mendelian randomization analysis of European prospective data. Int J Cancer.

[52] Wu PF, Li RZ, Zhang W, Hu HY, Wang W, Lin Y (2020). Polycystic ovary syndrome is causally associated with estrogen receptor-positive instead of estrogen receptor-negative breast cancer: a Mendelian randomization study. Am J Obstet Gynecol.

[53] Yarmolinsky J, Bull CJ, Walker VM, Nounu A, Davey Smith G (2020). Mendelian randomization applied to pharmaceutical use: the case of metformin and lung cancer. Int J Epidemiol.

[54] Li X, Meng X, Spiliopoulou A, Timofeeva M, Wei WQ, Gifford A, Shen X, He Y, Varley T, McKeigue P (2018). MR-PheWAS: exploring the causal effect of SUA level on multiple disease outcomes by using genetic instruments in UK Biobank. Ann Rheum Dis.

[55] Semmes EC, Vijayakrishnan J, Zhang C, Hurst JH, Houlston RS, Walsh KM (2020). Leveraging genome and phenome-wide association studies to investigate genetic risk of acute lymphoblastic leukemia. Cancer Epidemiol Biomark Prev.

[56] Schatzkin A, Abnet CC, Cross AJ, Gunter M, Pfeiffer R, Gail M, Lim U, Davey-Smith G (2009). Mendelian randomization: how it can--and cannot--help confirm causal relations between nutrition and cancer. Cancer Prev Res (Philadelphia, Pa).

[57] Allin KH, Nordestgaard BG (2011). Elevated C-reactive protein in the diagnosis, prognosis, and cause of cancer. Crit Rev Clin Lab Sci.

[58] Schutz FA, Pomerantz MM, Gray KP, Atkins MB, Rosenberg JE, Hirsch MS, McDermott DF, Lampron ME, Lee GS, Signoretti S (2013). Single nucleotide polymorphisms and risk of recurrence of renal-cell carcinoma: a cohort study. Lancet Oncol.

[59] Zgaga L, Agakov F, Theodoratou E, Farrington SM, Tenesa A, Dunlop MG, McKeigue P, Campbell H (2013). Model selection approach suggests causal association between 25-hydroxyvitamin D and colorectal cancer. PloS One.

[60] Yu H, Xu P, Cui Y (2016). Causal effect of vitamin D on prostate cancer using Mendelian randomization approach. World J Urol.

[61] Gage SH, Davey Smith G, Ware JJ, Flint J, Munafò MR (2016). G = E: what GWAS can tell us about the environment. PLoS Genet.

[62] O'Shea SJ, Davies JR, Newton-Bishop JA (2016). Vitamin D, vitamin A, the primary melanoma transcriptome and survival. Br J Dermatol.

[63] Franks PW, Atabaki-Pasdar N (2017). Causal inference in obesity research. J Int Med.

[64] Burgess S, Thompson DJ, Rees JMB, Day FR, Perry JR, Ong KK (2017). Dissecting causal pathways using Mendelian randomization with summarized genetic data: application to age at menarche and risk of breast cancer. Genetics.

[65] Kim Y, Kraft P, Asgari MM (2018). Using a Mendelian randomization approach to explore a causal relationship between vitamin D and nonmelanoma skin cancer. Br J Dermatol.

[66] Presley CJ, Tang D, Soulos PR, Chiang AC, Longtine JA, Adelson KB, Herbst RS, Zhu W, Nussbaum NC, Sorg RA (2018). Association of broad-based genomic sequencing with survival among patients with advanced non-small cell lung cancer in the community oncology setting. Jama.

[67] Muskens IS, Hansen HM, Smirnov IV, Molinaro AM, Bondy ML, Schildkraut JM, Wrensch M, Wiemels JL, Claus EB (2019). Longer genotypically-estimated leukocyte telomere length is associated with increased meningioma risk. J Neuro-oncol.

[68] Wu L, Shu X, Bao J, Guo X, Kote-Jarai Z, Haiman CA, Eeles RA, Zheng W (2019). Analysis of Over 140,000 European descendants identifies genetically predicted blood protein biomarkers associated with prostate cancer risk. Cancer Res.

[69] Jorde R, Wilsgaard T, Grimnes G (2019). Polymorphisms in the vitamin D system and mortality - the Tromsø study. J Steroid Biochem Mol Biol.

[70] Li X, Wu W, Giovannucci E, Stampfer MJ, Gao X, Han J (2020). Cutaneous nevi and internal cancer risk: Results from two large prospective cohorts of US women. Int J Cancer.

[71] Liu L, Zeng P, Yang S, Yuan Z (2020). Leveraging methylation to identify the potential causal genes associated with survival in lung adenocarcinoma and lung squamous cell carcinoma. Oncol Letters.

[72] Semple RK (2016). EJE PRIZE 2015: How does insulin resistance arise, and how does it cause disease? Human genetic lessons. Eur J Endocrinol.

[73] Zhang X, Gill D, He Y, Yang T, Li X, Monori G, Campbell H, Dunlop M, Tsilidis KK, Timofeeva M (2020). Non-genetic biomarkers and colorectal cancer risk: Umbrella review and evidence triangulation. Cancer Med.

[74] Sacerdote C, Guarrera S, Smith GD, Grioni S, Krogh V, Masala G, Mattiello A, Palli D, Panico S, Tumino R (2007). Lactase persistence and bitter taste response: instrumental variables and Mendelian randomization in epidemiologic studies of dietary factors and cancer risk. Am J Epidemiol.

[75] Liu YP, Gu YM, Thijs L, Knapen MH, Salvi E, Citterio L, Petit T, Carpini SD, Zhang Z, Jacobs L (2015). Inactive matrix Gla protein is causally related to adverse health outcomes: a Mendelian randomization study in a Flemish population. Hypertension (Dallas, Tex : 1979).

[76] Passarelli MN, Newcomb PA, Makar KW, Burnett-Hartman AN, Potter JD, Upton MP, Zhu LC, Rosenfeld ME, Schwartz SM, Rutter CM (2015). Blood lipids and colorectal polyps: testing an etiologic hypothesis using phenotypic measurements and Mendelian randomization. Cancer Causes Control : CCC.

[77] Borné Y, Smith JG, Nilsson PM, Melander O, Hedblad B, Engström G (2016). Total and differential leukocyte counts in relation to incidence of diabetes mellitus: a prospective population-based cohort study. PloS One.

[78] Ligthart S, Vaez A, Võsa U, Stathopoulou MG, de Vries PS, Prins BP, Van der Most PJ, Tanaka T, Naderi E, Rose LM (2018). Genome analyses of > 200,000 individuals identify 58 loci for chronic inflammation and highlight pathways that link inflammation and complex disorders. Am J Hum Genet.

[79] Isom CA, Shrubsole MJ, Cai Q, Smalley WE, Ness RM, Zheng W, Murff HJ (2019). Arachidonic acid and colorectal adenoma risk: a Mendelian randomization study. Clin Epidemiol.

[80] Sun YQ, Burgess S, Staley JR, Wood AM, Bell S, Kaptoge SK, Guo Q, Bolton TR, Mason AM, Butterworth AS (2019). Body mass index and all cause mortality in HUNT and UK Biobank studies: linear and non-linear Mendelian randomisation analyses. BMJ (Clin Res ed).

[81] Xu Z, Sandler DP, Taylor JA (2020). Blood DNA methylation and breast cancer: a prospective case-cohort analysis in the sister study. J Natl Cancer Inst.

[82] Zhou A, Morris HA, Hyppönen E (2019). Health effects associated with serum calcium concentrations: evidence from MR-PheWAS analysis in UK Biobank. Osteoporos Int.

[83] Mulugeta A, Zhou A, King C, Hyppönen E (2020). Association between major depressive disorder and multiple disease outcomes: a phenome-wide Mendelian randomisation study in the UK Biobank. Mol Psychiatr.

[84] Meng X, Li X, Timofeeva MN, He Y, Spiliopoulou A, Wei WQ, Gifford A, Wu H, Varley T, Joshi P (2019). Phenome-wide Mendelian-randomization study of genetically determined vitamin D on multiple health outcomes using the UK Biobank study. Int J Epidemiol.

[85] Seddighi S, Houck AL, Rowe JB, Pharoah PDP (2019). Evidence of a causal association between cancer and Alzheimer's disease: a Mendelian randomization analysis. Sci Rep.

[86] Li X, Meng X, He Y, Spiliopoulou A, Timofeeva M, Wei WQ, Gifford A, Yang T, Varley T, Tzoulaki I (2019). Genetically determined serum urate levels and cardiovascular and other diseases in UK Biobank cohort: a phenome-wide Mendelian randomization study. PLoS Med.

[87] Jayasuriya NA, Kjaergaard AD, Pedersen KM, Sørensen AL, Bak M, Larsen MK, Nordestgaard BG, Bojesen SE, Çolak Y, Skov V (2020). Smoking, blood cells and myeloproliferative neoplasms: meta-analysis and Mendelian randomization of 2·3 million people. Br J Haematol.

[88] Millard LAC, Munafò MR, Tilling K, Wootton RE, Davey Smith G (2019). MR-pheWAS with stratification and interaction: searching for the causal effects of smoking heaviness identified an effect on facial aging. PLoS Genet.

[89] Joshi PK, Pirastu N, Kentistou KA, Fischer K, Hofer E, Schraut KE, Clark DW, Nutile T, Barnes CLK, Timmers P (2017). Genome-wide meta-analysis associates HLA-DQA1/DRB1 and LPA and lifestyle factors with human longevity. Nat Commun.

[90] Huang R, Yanni S, CHAN KHK. The lung cancer associated MicroRNAs and single nucleotides polymorphisms: a Mendelian randomization analysis. In: 2020 42nd Annual International Conference of the IEEE Engineering in Medicine & Biology Society (EMBC). Piscataway: IEEE; 2020. p. 2346–52. 10.1109/EMBC44109.2020.9176344 33018478

[91] Thom CS, Voight BF (2020). Genetic colocalization atlas points to common regulatory sites and genes for hematopoietic traits and hematopoietic contributions to disease phenotypes. BMC Med Genomics.

[92] Nicolopoulos K, Mulugeta A, Zhou A, Hyppönen E (2020). Association between habitual coffee consumption and multiple disease outcomes: a Mendelian randomisation phenome-wide association study in the UK Biobank. Clin Nutr (Edinburgh, Scotland).

[93] Harrison S, Davies AR, Dickson M, Tyrrell J, Green MJ, Katikireddi SV, Campbell D, Munafò M, Dixon P, Jones HE (2020). The causal effects of health conditions and risk factors on social and socioeconomic outcomes: Mendelian randomization in UK Biobank. Int J Epidemiol.

[94] Lin HJ, Lakkides KM, Reddy ST, Louie AD, Kau IH, Zhou H, Gim JS, Ma HL, Matthies CF, Keku TO (2002). Prostaglandin H synthase 2 variant (Val511Ala) in African Americans may reduce the risk for colorectal neoplasia. Cancer Epidemiol Biomark Prev.

[95] Novotný L (2007). Bencko V: [Genotype-disease association and possibility to reveal environmentally modifiable disease causes: the use of Mendelian randomization principle]. Casopis lekaru ceskych.

[96] Lewis SJ, Smith GD (2005). Alcohol, ALDH2, and esophageal cancer: a meta-analysis which illustrates the potentials and limitations of a Mendelian randomization approach. Cancer Epidemiol Biomark Prev.

[97] Brennan P, Hsu CC, Moullan N, Szeszenia-Dabrowska N, Lissowska J, Zaridze D, Rudnai P, Fabianova E, Mates D, Bencko V (2005). Effect of cruciferous vegetables on lung cancer in patients stratified by genetic status: a Mendelian randomisation approach. Lancet (London, England).

[98] Boccia S, Hashibe M, Gallì P, De Feo E, Asakage T, Hashimoto T, Hiraki A, Katoh T, Nomura T, Yokoyama A (2009). Aldehyde dehydrogenase 2 and head and neck cancer: a meta-analysis implementing a Mendelian randomization approach. Cancer Epidemiol Biomark Prev.

[99] Brennan P, McKay J, Moore L, Zaridze D, Mukeria A, Szeszenia-Dabrowska N, Lissowska J, Rudnai P, Fabianova E, Mates D (2009). Obesity and cancer: Mendelian randomization approach utilizing the FTO genotype. Int J Epidemiol.

[100] Trompet S, Jukema JW, Katan MB, Blauw GJ, Sattar N, Buckley B, Caslake M, Ford I, Shepherd J, Westendorp RG (2009). Apolipoprotein e genotype, plasma cholesterol, and cancer: a Mendelian randomization study. Am J Epidemiol.

[101] Pierce BL, Ahsan H (2010). Genetic susceptibility to type 2 diabetes is associated with reduced prostate cancer risk. Hum Hered.

[102] Timpson NJ, Brennan P, Gaborieau V, Moore L, Zaridze D, Matveev V, Szeszenia-Dabrowska N, Lissowska J, Mates D, Bencko V (2010). Can lactase persistence genotype be used to reassess the relationship between renal cell carcinoma and milk drinking? Potentials and problems in the application of Mendelian randomization. Cancer Epidemiol Biomark Prev.

[103] Wang J, Wang H, Chen Y, Hao P, Zhang Y (2011). Alcohol ingestion and colorectal neoplasia: a meta-analysis based on a Mendelian randomization approach. Color Dis.

[104] Bonilla C, Gilbert R, Kemp JP, Timpson NJ, Evans DM, Donovan JL, Hamdy FC, Neal DE, Fraser WD, Davey SG (2013). Using genetic proxies for lifecourse sun exposure to assess the causal relationship of sun exposure with circulating vitamin d and prostate cancer risk. Cancer Epidemiol Biomark Prev.

[105] Tian Q, Jia J, Ling S, Liu Y, Yang S, Shao Z (2014). A causal role for circulating miR-34b in osteosarcoma. Eur J Surg Oncol.

[106] Davies JR, Field S, Randerson-Moor J, Harland M, Kumar R, Anic GM, Nagore E, Hansson J, Höiom V, Jönsson G (2014). An inherited variant in the gene coding for vitamin D-binding protein and survival from cutaneous melanoma: a BioGenoMEL study. Pigment Cell Melanoma Res.

[107] Song M, Gong J, Giovannucci EL, Berndt SI, Brenner H, Chang-Claude J, Curtis KR, Harrison TA, Hoffmeister M, Hsu L (2015). Genetic variants of adiponectin and risk of colorectal cancer. Int J Cancer.

[108] Trummer O, Langsenlehner U, Krenn-Pilko S, Pieber TR, Obermayer-Pietsch B, Gerger A, Renner W, Langsenlehner T (2016). Vitamin D and prostate cancer prognosis: a Mendelian randomization study. World J Urol.

[109] Davies NM, Gaunt TR, Lewis SJ, Holly J, Donovan JL, Hamdy FC, Kemp JP, Eeles R, Easton D, Kote-Jarai Z (2015). The effects of height and BMI on prostate cancer incidence and mortality: a Mendelian randomization study in 20,848 cases and 20,214 controls from the PRACTICAL consortium. Cancer Causes Control : CCC.

[110] Day FR, Ruth KS, Thompson DJ, Lunetta KL, Pervjakova N, Chasman DI, Stolk L, Finucane HK, Sulem P, Bulik-Sullivan B (2015). Large-scale genomic analyses link reproductive aging to hypothalamic signaling, breast cancer susceptibility and BRCA1-mediated DNA repair. Nat Genet.

[111] Thompson DJ, O'Mara TA, Glubb DM, Painter JN, Cheng T, Folkerd E, Doody D, Dennis J, Webb PM, Gorman M (2016). CYP19A1 fine-mapping and Mendelian randomization: estradiol is causal for endometrial cancer. Endocrine-related Cancer.

[112] Bull CJ, Bonilla C, Holly JM, Perks CM, Davies N, Haycock P, Yu OH, Richards JB, Eeles R, Easton D (2016). Blood lipids and prostate cancer: a Mendelian randomization analysis. Cancer Med.

[113] Brunner C, Davies NM, Martin RM, Eeles R, Easton D, Kote-Jarai Z, Al Olama AA, Benlloch S, Muir K, Giles G (2017). Alcohol consumption and prostate cancer incidence and progression: a Mendelian randomisation study. Int J Cancer.

[114] Taylor AE, Martin RM, Geybels MS, Stanford JL, Shui I, Eeles R, Easton D, Kote-Jarai Z (2017). Amin Al Olama A, Benlloch S et al: Investigating the possible causal role of coffee consumption with prostate cancer risk and progression using Mendelian randomization analysis. Int J Cancer.

[115] Lu WQ, Qiu JL, Huang ZL, Liu HY (2016). Enhanced circulating transforming growth factor beta 1 is causally associated with an increased risk of hepatocellular carcinoma: a Mendelian randomization meta-analysis. Oncotarget.

[116] Legason ID, Pfeiffer RM, Udquim KI, Bergen AW, Gouveia MH, Kirimunda S, Otim I, Karlins E, Kerchan P, Nabalende H (2017). Evaluating the causal link between malaria infection and endemic Burkitt lymphoma in Northern Uganda: a Mendelian randomization study. EBioMedicine.

[117] Bergholdt HKM, Nordestgaard BG, Varbo A, Ellervik C (2018). Lactase persistence, milk intake, and mortality in the Danish general population: a Mendelian randomization study. Eur J Epidemiol.

[118] Kobberø Lauridsen B, Stender S, Frikke-Schmidt R, Nordestgaard BG, Tybjærg-Hansen A (2017). Using genetics to explore whether the cholesterol-lowering drug ezetimibe may cause an increased risk of cancer. Int J Epidemiol.

[119] Gómez-Acebo I, Dierssen-Sotos T, Palazuelos C, Fernández-Navarro P, Castaño-Vinyals G, Alonso-Molero J, Urtiaga C, Fernández-Villa T, Ardanaz E, Rivas-Del-Fresno M (2018). Pigmentation phototype and prostate and breast cancer in a select Spanish population-a Mendelian randomization analysis in the MCC-Spain study. PloS One.

[120] Campa D, Matarazzi M, Greenhalf W, Bijlsma M, Saum KU, Pasquali C, van Laarhoven H, Szentesi A, Federici F, Vodicka P (2019). Genetic determinants of telomere length and risk of pancreatic cancer: a PANDoRA study. Int J Cancer.

[121] Vie G, Wootton RE, Bjørngaard JH, Åsvold BO, Taylor AE, Gabrielsen ME, Davey Smith G, Romundstad PR, Munafò MR (2019). The effect of smoking intensity on all-cause and cause-specific mortality-a Mendelian randomization analysis. Int J Epidemiol.

[122] Jung SY, Mancuso N, Papp J, Sobel E, Zhang ZF (2019). Post genome-wide gene-environment interaction study: the effect of genetically driven insulin resistance on breast cancer risk using Mendelian randomization. PloS One.

[123] Ibáñez-Sanz G, Díez-Villanueva A, Riera-Ponsati M, Fernández-Villa T, Fernández Navarro P, Bustamante M, Llorca J, Amiano P, Ascunce N, Fernández-Tardón G (2019). Mendelian randomization analysis rules out disylipidaemia as colorectal cancer cause. Sci Rep.

[124] Schmidt AF, Holmes MV, Preiss D, Swerdlow DI, Denaxas S, Fatemifar G, Faraway R, Finan C, Valentine D, Fairhurst-Hunter Z (2019). Phenome-wide association analysis of LDL-cholesterol lowering genetic variants in PCSK9. BMC Cardiovasc Disord.

[125] Xie SH, Fang R, Huang M, Dai J, Thrift AP, Anderson LA, Chow WH, Bernstein L, Gammon MD, Risch HA (2020). Association between levels of sex hormones and risk of esophageal adenocarcinoma and Barrett’s esophagus. Clin Gastroenterol Hepatol.

[126] Xu Y, Xu J, Chancoco H, Huang M, Torres KE, Gu J. Long leukocyte telomere length is associated with increased risks of soft tissue sarcoma: a Mendelian randomization study. Cancers. 2020:12(3). 10.3390/cancers12030594 PMC7139681 32150919

[127] Giaccherini M, Macauda A, Sgherza N, Sainz J, Gemignani F, Maldonado JMS, Jurado M, Tavano F, Mazur G, Jerez A (2020). Genetic polymorphisms associated with telomere length and risk of developing myeloproliferative neoplasms. Blood Cancer J.

[128] Iles MM, Bishop DT, Taylor JC, Hayward NK, Brossard M, Cust AE, et al. The effect on melanoma risk of genes previously associated with telomere length. J Natl Cancer Inst. 2014;106:–10. 10.1093/jnci/dju267 PMC4196080 25231748

[129] Du J, Zhu X, Xie C, Dai N, Gu Y, Zhu M, Wang C, Gao Y, Pan F, Ren C (2015). Telomere length, genetic variants and gastric cancer risk in a Chinese population. Carcinogenesis.

[130] Prescott J, Setiawan VW, Wentzensen N, Schumacher F, Yu H, Delahanty R, Bernstein L, Chanock SJ, Chen C, Cook LS (2015). Body mass index genetic risk score and endometrial cancer risk. PloS One.

[131] Ojha J, Codd V, Nelson CP, Samani NJ, Smirnov IV, Madsen NR, Hansen HM, de Smith AJ, Bracci PM, Wiencke JK (2016). Genetic variation associated with longer telomere length increases risk of chronic lymphocytic leukemia. Cancer Epidemiol Biomark Prev.

[132] Walsh KM, Whitehead TP, de Smith AJ, Smirnov IV, Park M, Endicott AA, Francis SS, Codd V, Samani NJ, Metayer C (2016). Common genetic variants associated with telomere length confer risk for neuroblastoma and other childhood cancers. Carcinogenesis.

[133] Rode L, Nordestgaard BG, Bojesen SE (2016). Long telomeres and cancer risk among 95 568 individuals from the general population. Int J Epidemiol.

[134] Luu HN, Long J, Wen W, Zheng Y, Cai Q, Gao YT, Zheng W, Shu XO (2016). Association between genetic risk score for telomere length and risk of breast cancer. Cancer Causes Control : CCC.

[135] Antwi SO, Bamlet WR, Broderick BT, Chaffee KG, Oberg A, Jatoi A, Boardman LA, Petersen GM (2017). Genetically predicted telomere length is not associated with pancreatic cancer risk. Cancer Epidemiol Biomark Prev.

[136] Howell AE, Zheng J, Haycock PC, McAleenan A, Relton C, Martin RM, Kurian KM (2018). Use of Mendelian randomization for identifying risk factors for brain tumors. Frontiers in genetics.

[137] Gentiluomo M, Canzian F, Nicolini A, Gemignani F, Landi S, Campa D. Germline genetic variability in pancreatic cancer risk and prognosis. Sem Cancer Biol. 2020. 10.1016/j.semcancer.2020.08.003 32818625

[138] Fussey JM, Beaumont RN, Wood AR, Vaidya B, Smith J, Tyrrell J (2020). Mendelian randomization supports a causative effect of TSH on thyroid carcinoma. Endocrine-related Cancer.

[139] Allin KH, Nordestgaard BG, Zacho J, Tybjaerg-Hansen A, Bojesen SE (2010). C-reactive protein and the risk of cancer: a Mendelian randomization study. J Natl Cancer Inst.

[140] Benn M, Tybjærg-Hansen A, Stender S, Frikke-Schmidt R, Nordestgaard BG (2011). Low-density lipoprotein cholesterol and the risk of cancer: a Mendelian randomization study. J Natl Cancer Inst.

[141] Collin SM, Metcalfe C, Palmer TM, Refsum H, Lewis SJ, Smith GD, Cox A, Davis M, Marsden G, Johnston C (2011). The causal roles of vitamin B(12) and transcobalamin in prostate cancer: can Mendelian randomization analysis provide definitive answers?. Int J Mol Epidemiol Genetics.

[142] Heikkilä K, Silander K, Salomaa V, Jousilahti P, Koskinen S, Pukkala E, et al. C-reactive protein-associated genetic variants and cancer risk: findings from FINRISK 1992, FINRISK 1997 and Health 2000 studies. Eur J Cancer (Oxford, England : 1990) 2011. 47(3):404–12. 10.1016/j.ejca.2010.07.032 20727736

[143] Theodoratou E, Palmer T, Zgaga L, Farrington SM, McKeigue P, Din FV, Tenesa A, Davey-Smith G, Dunlop MG, Campbell H (2012). Instrumental variable estimation of the causal effect of plasma 25-hydroxy-vitamin D on colorectal cancer risk: a Mendelian randomization analysis. PloS one.

[144] Afzal S, Brøndum-Jacobsen P, Bojesen SE, Nordestgaard BG: Genetically low vitamin D concentrations and increased mortality: Mendelian randomisation analysis in three large cohorts. BMJ (Clin Res ed) 2014, 349:g6330. 10.1136/bmj.g6330 PMC4238742 25406188

[145] Thrift AP, Risch HA, Onstad L, Shaheen NJ, Casson AG, Bernstein L, Corley DA, Levine DM, Chow WH, Reid BJ (2014). Risk of esophageal adenocarcinoma decreases with height, based on consortium analysis and confirmed by Mendelian randomization. Clin Gastroenterol Hepatol.

[146] Thrift AP, Shaheen NJ, Gammon MD, Bernstein L, Reid BJ, Onstad L, Risch HA, Liu G, Bird NC, Wu AH (2014). Obesity and risk of esophageal adenocarcinoma and Barrett's esophagus: a Mendelian randomization study. J Natl Cancer Inst.

[147] Wium-Andersen MK, Orsted DD, Nordestgaard BG (2014). Elevated C-reactive protein, depression, somatic diseases, and all-cause mortality: a mendelian randomization study. Biol Psychiatr.

[148] Kjaergaard AD, Nordestgaard BG, Johansen JS, Bojesen SE (2015). Observational and genetic plasma YKL-40 and cancer in 96,099 individuals from the general population. Int J Cancer.

[149] Nead KT, Sharp SJ, Thompson DJ, Painter JN, Savage DB, Semple RK, Barker A, Perry JR, Attia J, Dunning AM (2015). Evidence of a causal association between insulinemia and endometrial cancer: a Mendelian randomization analysis. J Natl Cancer Inst.

[150] Nimptsch K, Aleksandrova K, Boeing H, Janke J, Lee YA, Jenab M, Bueno-de-Mesquita HB, Jansen EH, Tsilidis KK, Trichopoulou A (2015). Association of CRP genetic variants with blood concentrations of C-reactive protein and colorectal cancer risk. Int J Cancer.

[151] Nimptsch K, Aleksandrova K, Boeing H, Janke J, Lee YA, Jenab M, Kong SY, Tsilidis KK, Weiderpass E, Bueno-De-Mesquita HB (2015). Plasma fetuin-a concentration, genetic variation in the AHSG gene and risk of colorectal cancer. Int J Cancer.

[152] Pei Y, Xu Y, Niu W (2015). Causal relevance of circulating adiponectin with cancer: a meta-analysis implementing Mendelian randomization. Tumour Biol.

[153] Rode L, Nordestgaard BG, Bojesen SE (2015). Peripheral blood leukocyte telomere length and mortality among 64,637 individuals from the general population. J Natl Cancer Inst.

[154] Thrift AP, Gong J, Peters U, Chang-Claude J, Rudolph A, Slattery ML, Chan AT, Esko T, Wood AR, Yang J (2015). Mendelian randomization study of height and risk of colorectal cancer. Int J Epidemiol.

[155] Thrift AP, Gong J, Peters U, Chang-Claude J, Rudolph A, Slattery ML, Chan AT, Locke AE, Kahali B, Justice AE (2015). Mendelian randomization study of body mass index and colorectal cancer risk. Cancer Epidemiol Biomark Prev.

[156] Tian G, Mi J, Wei X, Zhao D, Qiao L, Yang C, Li X, Zhang S, Li X, Wang B (2015). Circulating interleukin-6 and cancer: a meta-analysis using Mendelian randomization. Sci Rep.

[157] Walsh KM, Codd V, Rice T, Nelson CP, Smirnov IV, McCoy LS, Hansen HM, Elhauge E, Ojha J, Francis SS (2015). Longer genotypically-estimated leukocyte telomere length is associated with increased adult glioma risk. Oncotarget.

[158] Yang C, Tian G, Mi J, Wei X, Li X, Li X, Wang W, Wang B (2015). Causal relevance of circulating high-density lipoprotein cholesterol with cancer: a Mendelian randomization meta-analysis. Sci Rep.

[159] Zhang B, Shu XO, Delahanty RJ, Zeng C, Michailidou K, Bolla MK, et al. Height and breast cancer risk: evidence from prospective studies and Mendelian randomization. J Natl Cancer Inst. 2015:107(11). 10.1093/jnci/djv219 PMC4643630 26296642

[160] Zhang C, Doherty JA, Burgess S, Hung RJ, Lindström S, Kraft P, Gong J, Amos CI, Sellers TA, Monteiro AN (2015). Genetic determinants of telomere length and risk of common cancers: a Mendelian randomization study. Hum Mol Genet.

[161] Benn M, Tybjærg-Hansen A, Smith GD, Nordestgaard BG (2016). High body mass index and cancer risk-a Mendelian randomisation study. Eur J Epidemiol.

[162] Carreras-Torres R, Haycock PC, Relton CL, Martin RM, Smith GD, Kraft P, Gao C, Tworoger S, Le Marchand L, Wilkens LR (2016). The causal relevance of body mass index in different histological types of lung cancer: a Mendelian randomization study. Sci Rep.

[163] Dixon SC, Nagle CM, Thrift AP, Pharoah PD, Pearce CL, Zheng W, Painter JN, Chenevix-Trench G, Fasching PA, Beckmann MW (2016). Adult body mass index and risk of ovarian cancer by subtype: a Mendelian randomization study. Int J Epidemiol.

[164] Gao C, Patel CJ, Michailidou K, Peters U, Gong J, Schildkraut J, Schumacher FR, Zheng W, Boffetta P, Stucker I (2016). Mendelian randomization study of adiposity-related traits and risk of breast, ovarian, prostate, lung and colorectal cancer. Int J Epidemiol.

[165] Guo Y, Warren Andersen S, Shu XO, Michailidou K, Bolla MK, Wang Q, Garcia-Closas M, Milne RL, Schmidt MK, Chang-Claude J (2016). Genetically predicted body mass index and breast cancer risk: Mendelian randomization analyses of data from 145,000 women of European descent. PLoS Med.

[166] Huang Q, Mi J, Wang X, Liu F, Wang D, Yan D, Wang B, Zhang S, Tian G (2016). Genetically lowered concentrations of circulating sRAGE might cause an increased risk of cancer: meta-analysis using Mendelian randomization. J Int Med Res.

[167] Jarvis D, Mitchell JS, Law PJ, Palin K, Tuupanen S, Gylfe A, Hänninen UA, Cajuso T, Tanskanen T, Kondelin J (2016). Mendelian randomisation analysis strongly implicates adiposity with risk of developing colorectal cancer. Br J Cancer.

[168] Khankari NK, Murff HJ, Zeng C, Wen W, Eeles RA, Easton DF, Kote-Jarai Z, Al Olama AA, Benlloch S, Muir K (2016). Polyunsaturated fatty acids and prostate cancer risk: a Mendelian randomisation analysis from the PRACTICAL consortium. Br J Cancer.

[169] Khankari NK, Shu XO, Wen W, Kraft P, Lindström S, Peters U, Schildkraut J, Schumacher F, Bofetta P, Risch A (2016). Association between adult height and risk of colorectal, lung, and prostate cancer: results from meta-analyses of prospective studies and Mendelian randomization analyses. PLoS Med.

[170] Machiela MJ, Lan Q, Slager SL, Vermeulen RC, Teras LR, Camp NJ, Cerhan JR, Spinelli JJ, Wang SS, Nieters A (2016). Genetically predicted longer telomere length is associated with increased risk of B-cell lymphoma subtypes. Hum Mol Genet.

[171] Niu W, Pang Q, Lin T, Wang Z, Zhang J, Tai M, Zhang L, Zhang L, Gu M, Liu C (2016). A causal role of genetically elevated circulating interleukin-10 in the development of digestive cancers: evidence from Mendelian randomization analysis based on 29,307 subjects. Medicine.

[172] Ong JS, Cuellar-Partida G, Lu Y, Fasching PA, Hein A, Burghaus S, Beckmann MW, Lambrechts D, Van Nieuwenhuysen E, Vergote I (2016). Association of vitamin D levels and risk of ovarian cancer: a Mendelian randomization study. Int J Epidemiol.

[173] Painter JN, O'Mara TA, Marquart L, Webb PM, Attia J, Medland SE, Cheng T, Dennis J, Holliday EG, McEvoy M (2016). Genetic risk score Mendelian randomization shows that obesity measured as body mass index, but not waist:hip ratio, is causal for endometrial cancer. Cancer Epidemiol Biomark Prev.

[174] Qu K, Pang Q, Lin T, Zhang L, Gu M, Niu W, Liu C, Zhang M (2016). Circulating interleukin-10 levels and human papilloma virus and Epstein-Barr virus-associated cancers: evidence from a Mendelian randomization meta-analysis based on 11,170 subjects. OncoTargets Therapy.

[175] Xu W, Cheng Y, Zhu H (2016). Evaluation of an association of blood homocysteine levels with gastric cancer risk from 27 case-control studies. Medicine.

[176] Xuan Y, Li XH, Hu ZQ, Teng ZM, Hu DJ (2016). A Mendelian randomization study of plasma homocysteine and multiple myeloma. Sci Rep.

[177] Carreras-Torres R, Johansson M, Gaborieau V, Haycock PC, Wade KH, Relton CL, Martin RM, Davey Smith G, Brennan P (2017). The role of obesity, type 2 diabetes, and metabolic factors in pancreatic cancer: a Mendelian randomization study. J Natl Cancer Inst.

[178] Carreras-Torres R, Johansson M, Haycock PC, Wade KH, Relton CL, Martin RM, Davey Smith G, Albanes D, Aldrich MC, Andrew A (2017). Obesity, metabolic factors and risk of different histological types of lung cancer: a Mendelian randomization study. PloS One.

[179] Cheng Y, Yu C, Huang M, Du F, Song C, Ma Z, Zhai X, Yang Y, Liu J, Bei JX (2017). Genetic association of telomere length with hepatocellular carcinoma risk: a Mendelian randomization analysis. Cancer Epidemiol.

[180] Day FR, Thompson DJ, Helgason H, Chasman DI, Finucane H, Sulem P, Ruth KS, Whalen S, Sarkar AK, Albrecht E (2017). Genomic analyses identify hundreds of variants associated with age at menarche and support a role for puberty timing in cancer risk. Nat Genet.

[181] Dimitrakopoulou VI, Tsilidis KK, Haycock PC, Dimou NL, Al-Dabhani K, Martin RM, Lewis SJ, Gunter MJ, Mondul A, Shui IM (2017). Circulating vitamin D concentration and risk of seven cancers: Mendelian randomisation study. BMJ (Clin Res ed).

[182] Haycock PC, Burgess S, Nounu A, Zheng J, Okoli GN, Bowden J, Wade KH, Timpson NJ, Evans DM, Willeit P (2017). Association between telomere length and risk of cancer and non-neoplastic diseases: a Mendelian randomization study. JAMA Oncol.

[183] Kobylecki CJ, Afzal S, Nordestgaard BG (2017). Plasma urate, cancer incidence, and all-cause mortality: a Mendelian randomization study. Clin Chem.

[184] Levy M, Hall D, Sud A, Law P, Litchfield K, Dudakia D, Haugen TB, Karlsson R, Reid A, Huddart RA (2017). Mendelian randomisation analysis provides no evidence for a relationship between adult height and testicular cancer risk. Andrology.

[185] Machiela MJ, Hofmann JN, Carreras-Torres R, Brown KM, Johansson M, Wang Z, Foll M, Li P, Rothman N, Savage SA (2017). Genetic variants related to longer telomere length are associated with increased risk of renal cell carcinoma. Eur Urol.

[186] Mao Y, Yan C, Lu Q, Zhu M, Yu F, Wang C, Dai J, Ma H, Hu Z, Shen H (2017). Genetically predicted high body mass index is associated with increased gastric cancer risk. Eur J Hum Genet EJHG.

[187] May-Wilson S, Sud A, Law PJ, Palin K, Tuupanen S, Gylfe A, Hänninen UA, Cajuso T, Tanskanen T, Kondelin J (2017). Pro-inflammatory fatty acid profile and colorectal cancer risk: a Mendelian randomisation analysis. Eur J Cancer (Oxford, England : 1990).

[188] Nimptsch K, Song M, Aleksandrova K, Katsoulis M, Freisling H, Jenab M, Gunter MJ, Tsilidis KK, Weiderpass E, Bueno-De-Mesquita HB (2017). Genetic variation in the ADIPOQ gene, adiponectin concentrations and risk of colorectal cancer: a Mendelian Randomization analysis using data from three large cohort studies. Eur J Epidemiol.

[189] Rodriguez-Broadbent H, Law PJ, Sud A, Palin K, Tuupanen S, Gylfe A, Hänninen UA, Cajuso T, Tanskanen T, Kondelin J (2017). Mendelian randomisation implicates hyperlipidaemia as a risk factor for colorectal cancer. Int J Cancer.

[190] Wang C, Qin N, Zhu M, Chen M, Xie K, Cheng Y, Dai J, Liu J, Xia Y, Ma H (2017). Metabolome-wide association study identified the association between a circulating polyunsaturated fatty acids variant rs174548 and lung cancer. Carcinogenesis.

[191] Chandler PD, Tobias DK, Wang L, Smith-Warner SA, Chasman DI, Rose L, Giovannucci EL, Buring JE, Ridker PM, Cook NR (2018). Association between vitamin D genetic risk score and cancer risk in a large cohort of U.S. women. Nutrients.

[192] Disney-Hogg L, Cornish AJ, Sud A, Law PJ, Kinnersley B, Jacobs DI, Ostrom QT, Labreche K, Eckel-Passow JE, Armstrong GN (2018). Impact of atopy on risk of glioma: a Mendelian randomisation study. BMC Med.

[193] Disney-Hogg L, Sud A, Law PJ, Cornish AJ, Kinnersley B, Ostrom QT, Labreche K, Eckel-Passow JE, Armstrong GN, Claus EB (2018). Influence of obesity-related risk factors in the aetiology of glioma. Br J Cancer.

[194] Dixon-Suen SC, Nagle CM, Thrift AP, Pharoah PDP, Ewing A, Pearce CL, Zheng W, Chenevix-Trench G, Fasching PA, Beckmann MW (2018). Adult height is associated with increased risk of ovarian cancer: a Mendelian randomisation study. Br J Cancer.

[195] Doherty A, Smith-Byrne K, Ferreira T, Holmes MV, Holmes C, Pulit SL, Lindgren CM (2018). GWAS identifies 14 loci for device-measured physical activity and sleep duration. Nat Commun.

[196] Dudding T, Johansson M, Thomas SJ, Brennan P, Martin RM, Timpson NJ (2018). Assessing the causal association between 25-hydroxyvitamin D and the risk of oral and oropharyngeal cancer using Mendelian randomization. Int J Cancer.

[197] He L, Culminskaya I, Loika Y, Arbeev KG, Bagley O, Duan M, Yashin AI, Kulminski AM (2018). Causal effects of cardiovascular risk factors on onset of major age-related diseases: a time-to-event Mendelian randomization study. Exp Gerontol.

[198] He Y, Timofeeva M, Farrington SM, Vaughan-Shaw P, Svinti V, Walker M, Zgaga L, Meng X, Li X, Spiliopoulou A (2018). Exploring causality in the association between circulating 25-hydroxyvitamin D and colorectal cancer risk: a large Mendelian randomisation study. BMC Med.

[199] Lai FY, Nath M, Hamby SE, Thompson JR, Nelson CP, Samani NJ (2018). Adult height and risk of 50 diseases: a combined epidemiological and genetic analysis. BMC Med.

[200] Liyanage UE, Law MH, Ong JS, Cust AE, Mann GJ, Ward SV, Gharahkhani P, Iles MM, MacGregor S (2018). Polyunsaturated fatty acids and risk of melanoma: a Mendelian randomisation analysis. Int J Cancer.

[201] Neumeyer S, Banbury BL, Arndt V, Berndt SI, Bezieau S, Bien SA, Buchanan DD, Butterbach K, Caan BJ, Campbell PT (2018). Mendelian randomisation study of age at menarche and age at menopause and the risk of colorectal cancer. Br J Cancer.

[202] Nowak C, Ärnlöv J (2018). A Mendelian randomization study of the effects of blood lipids on breast cancer risk. Nat Commun.

[203] O'Mara TA, Glubb DM, Amant F, Annibali D, Ashton K, Attia J, Auer PL, Beckmann MW, Black A, Bolla MK (2018). Identification of nine new susceptibility loci for endometrial cancer. Nat Commun.

[204] Ong JS, An J, Law MH, Whiteman DC, Neale RE, Gharahkhani P, MacGregor S (2018). Height and overall cancer risk and mortality: evidence from a Mendelian randomisation study on 310,000 UK Biobank participants. Br J Cancer.

[205] Ong JS, Gharahkhani P, An J, Law MH, Whiteman DC, Neale RE, MacGregor S (2018). Vitamin D and overall cancer risk and cancer mortality: a Mendelian randomization study. Hum Mol Genet.

[206] Ong JS, Hwang LD, Cuellar-Partida G, Martin NG, Chenevix-Trench G, Quinn MCJ, Cornelis MC, Gharahkhani P, Webb PM, MacGregor S (2018). Assessment of moderate coffee consumption and risk of epithelial ovarian cancer: a Mendelian randomization study. Int J Epidemiol.

[207] Orho-Melander M, Hindy G, Borgquist S, Schulz CA, Manjer J, Melander O, Stocks T (2018). Blood lipid genetic scores, the HMGCR gene and cancer risk: a Mendelian randomization study. Int J Epidemiol.

[208] Pastorino R, Puggina A, Carreras-Torres R, Lagiou P, Holcátová I, Richiardi L, Kjaerheim K, Agudo A, Castellsagué X, Macfarlane TV (2018). Genetic contributions to the association between adult height and head and neck cancer: a Mendelian randomization analysis. Sci Rep.

[209] Rachakonda S, Kong H, Srinivas N, Garcia-Casado Z, Requena C, Fallah M, Heidenreich B, Planelles D, Traves V, Schadendorf D (2018). Telomere length, telomerase reverse transcriptase promoter mutations, and melanoma risk. Genes Chromosomes Cancer.

[210] Shi J, Wu L, Zheng W, Wen W, Wang S, Shu X, Long J, Shen CY, Wu PE, Saloustros E (2018). Genetic evidence for the association between schizophrenia and breast cancer. J Psychiatr Brain Sci.

[211] Sun YQ, Brumpton BM, Bonilla C, Lewis SJ, Burgess S, Skorpen F, Chen Y, Nilsen TIL, Romundstad PR, Mai XM (2018). Serum 25-hydroxyvitamin D levels and risk of lung cancer and histologic types: a Mendelian randomisation analysis of the HUNT study. Eur Respir J.

[212] Takahashi H, Cornish AJ, Sud A, Law PJ, Kinnersley B, Ostrom QT, Labreche K, Eckel-Passow JE, Armstrong GN, Claus EB (2018). Mendelian randomisation study of the relationship between vitamin D and risk of glioma. Sci Rep.

[213] Tan VY, Biernacka KM, Dudding T, Bonilla C, Gilbert R, Kaplan RC, Qibin Q, Teumer A, Martin RM, Perks CM (2018). Reassessing the association between circulating vitamin D and IGFBP-3: observational and Mendelian randomization estimates from independent sources. Cancer Epidemiol Biomark Prev.

[214] Wade KH, Carslake D, Sattar N, Davey Smith G, Timpson NJ (2018). BMI and mortality in UK Biobank: revised estimates using Mendelian randomization. Obesity (Silver Spring, Md).

[215] Wang L, Huang M, Ding H, Jin G, Chen L, Chen F, Shen H (2018). Genetically determined height was associated with lung cancer risk in East Asian population. Cancer Med.

[216] Wang S, Huo D, Kupfer S, Alleyne D, Ojengbede O, Zheng W, Nathanson KL, Nemesure B, Ambs S, Ogundiran TO (2018). Genetic variation in the vitamin D related pathway and breast cancer risk in women of African ancestry in the root consortium. Int J Cancer.

[217] Winsløw UC, Nordestgaard BG, Afzal S (2018). High plasma 25-hydroxyvitamin D and high risk of nonmelanoma skin cancer: a Mendelian randomization study of 97849 individuals. Br J Dermatol.

[218] Yarmolinsky J, Berryman K, Langdon R, Bonilla C, Davey Smith G, Martin RM, Lewis SJ (2018). Mendelian randomization does not support serum calcium in prostate cancer risk. Cancer Causes Control : CCC.

[219] Yarmolinsky J, Bonilla C, Haycock PC, Langdon RJQ, Lotta LA, Langenberg C, Relton CL, Lewis SJ, Evans DM, Davey Smith G (2018). Circulating selenium and prostate cancer risk: a Mendelian randomization analysis. J Natl Cancer Inst.

[220] Zhang C, Morimoto LM, de Smith AJ, Hansen HM, Gonzalez-Maya J, Endicott AA, Smirnov IV, Metayer C, Wei Q, Eward WC (2018). Genetic determinants of childhood and adult height associated with osteosarcoma risk. Cancer.

[221] Adams CD, Neuhausen SL (2019). Bi-directional Mendelian randomization of epithelial ovarian cancer and schizophrenia and uni-directional Mendelian randomization of schizophrenia on circulating 1- or 2-glycerophosphocholine metabolites. Mol Genet Metabolism Rep.

[222] Adams CD, Richmond R, Ferreira DLS, Spiller W, Tan V, Zheng J, Würtz P, Donovan J, Hamdy F, Neal D (2019). Circulating metabolic biomarkers of screen-detected prostate cancer in the ProtecT study. Cancer Epidemiol Biomark Prev.

[223] Au Yeung SL, Luo S, Schooling CM (2019). The impact of GDF-15, a biomarker for metformin, on the risk of coronary artery disease, breast and colorectal cancer, and type 2 diabetes and metabolic traits: a Mendelian randomisation study. Diabetologia.

[224] Au Yeung SL, Schooling CM (2019). Impact of glycemic traits, type 2 diabetes and metformin use on breast and prostate cancer risk: a Mendelian randomization study. BMJ Open Diabetes Res Care.

[225] Battram T, Richmond RC, Baglietto L, Haycock PC, Perduca V, Bojesen SE, Gaunt TR, Hemani G, Guida F, Carreras-Torres R (2019). Appraising the causal relevance of DNA methylation for risk of lung cancer. Int J Epidemiol.

[226] Beynon RA, Richmond RC, Santos Ferreira DL, Ness AR, May M, Smith GD, Vincent EE, Adams C, Ala-Korpela M, Würtz P (2019). Investigating the effects of lycopene and green tea on the metabolome of men at risk of prostate cancer: the ProDiet randomised controlled trial. Int J Cancer.

[227] Byrne EM, Ferreira MAR, Xue A, Lindström S, Jiang X, Yang J, Easton DF, Wray NR, Chenevix-Trench G (2019). Is schizophrenia a risk factor for breast cancer?-Evidence from genetic data. Schizophrenia Bull.

[228] Cao X, Huang M, Zhu M, Fang R, Ma Z, Jiang T, Dai J, Ma H, Jin G, Shen H (2019). Mendelian randomization study of telomere length and lung cancer risk in East Asian population. Cancer Med.

[229] Censin JC, Peters SAE, Bovijn J, Ferreira T, Pulit SL, Mägi R, Mahajan A, Holmes MV, Lindgren CM (2019). Causal relationships between obesity and the leading causes of death in women and men. PLoS Genet.

[230] Dashti HS, Merino J, Lane JM, Song Y, Smith CE, Tanaka T, McKeown NM, Tucker C, Sun D, Bartz TM (2019). Genome-wide association study of breakfast skipping links clock regulation with food timing. Am J Clin Nutr.

[231] Dimou NL, Papadimitriou N, Gill D, Christakoudi S, Murphy N, Gunter MJ, Travis RC, Key TJ, Fortner RT, Haycock PC (2019). Sex hormone binding globulin and risk of breast cancer: a Mendelian randomization study. Int J Epidemiol.

[232] Dong J, Gharahkhani P, Chow WH, Gammon MD, Liu G, Caldas C, Wu AH, Ye W, Onstad L, Anderson LA (2019). No association between vitamin D status and risk of Barrett’s esophagus or esophageal adenocarcinoma: a Mendelian randomization study. Clin Gastroenterol Hepatol.

[233] Fanidi A, Carreras-Torres R, Larose TL, Yuan JM, Stevens VL, Weinstein SJ, Albanes D, Prentice R, Pettinger M, Cai Q (2019). Is high vitamin B12 status a cause of lung cancer?. Int J Cancer.

[234] Ference BA, Ray KK, Catapano AL, Ference TB, Burgess S, Neff DR, Oliver-Williams C, Wood AM, Butterworth AS, Di Angelantonio E (2019). Mendelian Randomization study of ACLY and cardiovascular disease. N Engl J Med.

[235] Gallagher CS, Mäkinen N, Harris HR, Rahmioglu N, Uimari O, Cook JP, Shigesi N, Ferreira T, Velez-Edwards DR, Edwards TL (2019). Genome-wide association and epidemiological analyses reveal common genetic origins between uterine leiomyomata and endometriosis. Nat Commun.

[236] Gharahkhani P, Ong JS, An J, Law MH, Whiteman DC, Neale RE, MacGregor S (2019). Effect of increased body mass index on risk of diagnosis or death from cancer. Br J Cancer.

[237] Harris HR, Cushing-Haugen KL, Webb PM, Nagle CM, Jordan SJ, Risch HA, Rossing MA, Doherty JA, Goodman MT, Modugno F (2019). Association between genetically predicted polycystic ovary syndrome and ovarian cancer: a Mendelian randomization study. Int J Epidemiol.

[238] Huang T, Afzal S, Yu C, Guo Y, Bian Z, Yang L, Millwood IY, Walters RG, Chen Y, Chen N (2019). Vitamin D and cause-specific vascular disease and mortality: a Mendelian randomisation study involving 99,012 Chinese and 106,911 European adults. BMC Med.

[239] Jiang X, Dimou NL, Al-Dabhani K, Lewis SJ, Martin RM, Haycock PC, Gunter MJ, Key TJ, Eeles RA, Muir K (2019). Circulating vitamin D concentrations and risk of breast and prostate cancer: a Mendelian randomization study. Int J Epidemiol.

[240] Johansson M, Carreras-Torres R, Scelo G, Purdue MP, Mariosa D, Muller DC, Timpson NJ, Haycock PC, Brown KM, Wang Z (2019). The influence of obesity-related factors in the etiology of renal cell carcinoma-a Mendelian randomization study. PLoS Med.

[241] Kachuri L, Saarela O, Bojesen SE, Davey Smith G, Liu G, Landi MT, Caporaso NE, Christiani DC, Johansson M, Panico S (2019). Mendelian randomization and mediation analysis of leukocyte telomere length and risk of lung and head and neck cancers. Int J Epidemiol.

[242] Kar SP, Andrulis IL, Brenner H, Burgess S, Chang-Claude J, Considine D, Dörk T, Evans DGR, Gago-Domínguez M, Giles GG (2019). The association between weight at birth and breast cancer risk revisited using Mendelian randomisation. Eur J Epidemiol.

[243] Kho PF, Glubb DM, Thompson DJ, Spurdle AB, O'Mara TA (2019). Assessing the role of selenium in endometrial cancer risk: a Mendelian randomization study. Front Oncol.

[244] Kuo CL, Pilling LC, Kuchel GA, Ferrucci L, Melzer D (2019). Telomere length and aging-related outcomes in humans: a Mendelian randomization study in 261,000 older participants. Aging cell.

[245] Langdon RJ, Richmond RC, Hemani G, Zheng J, Wade KH, Carreras-Torres R, Johansson M, Brennan P, Wootton RE, Munafo MR (2019). A phenome-wide Mendelian randomization study of pancreatic cancer using summary genetic data. Cancer Epidemiol Biomark Prev.

[246] Li M, Kwok MK, Fong SSM, Schooling CM (2019). Indoleamine 2,3-dioxygenase and ischemic heart disease: a Mendelian randomization study. Sci Rep.

[247] Liu J, Zhou H, Zhang Y, Huang Y, Fang W, Yang Y, Hong S, Chen G, Zhao S, Chen X (2019). Docosapentaenoic acid and lung cancer risk: a Mendelian randomization study. Cancer Med.

[248] Liyanage UE, Ong JS, An J, Gharahkhani P, Law MH, MacGregor S (2019). Mendelian randomization study for genetically predicted polyunsaturated fatty acids levels on overall cancer risk and mortality. Cancer Epidemiol Biomark Prev.

[249] Ong JS, Law MH, An J, Han X, Gharahkhani P, Whiteman DC, Neale RE, MacGregor S (2019). Association between coffee consumption and overall risk of being diagnosed with or dying from cancer among > 300000 UK Biobank participants in a large-scale Mendelian randomization study. Int J Epidemiol.

[250] Ooi BNS, Loh H, Ho PJ, Milne RL, Giles G, Gao C, Kraft P, John EM, Swerdlow A, Brenner H (2019). The genetic interplay between body mass index, breast size and breast cancer risk: a Mendelian randomization analysis. Int J Epidemiol.

[251] Qian F, Rookus MA, Leslie G, Risch HA, Greene MH, Aalfs CM, Adank MA, Adlard J, Agnarsson BA, Ahmed M (2019). Mendelian randomisation study of height and body mass index as modifiers of ovarian cancer risk in 22,588 BRCA1 and BRCA2 mutation carriers. Br J Cancer.

[252] Qian F, Wang S, Mitchell J, McGuffog L, Barrowdale D, Leslie G, Oosterwijk JC, Chung WK, Evans DG, Engel C (2019). Height and body mass index as modifiers of breast cancer risk in BRCA1/2 mutation carriers: a Mendelian randomization study. J Natl Cancer Inst.

[253] Qin N, Li N, Wang C, Pu Z, Ma Z, Jin G, Zhu M, Dai M, Hu Z, Ma H (2019). Association of mosaic loss of chromosome Y with lung cancer risk and prognosis in a Chinese population. J Thoracic Oncol.

[254] Richmond RC, Anderson EL, Dashti HS, Jones SE, Lane JM, Strand LB, Brumpton B, Rutter MK, Wood AR, Straif K (2019). Investigating causal relations between sleep traits and risk of breast cancer in women: Mendelian randomisation study. BMJ (Clin Res ed).

[255] Shu X, Wu L, Khankari NK, Shu XO, Wang TJ, Michailidou K, Bolla MK, Wang Q, Dennis J, Milne RL (2019). Associations of obesity and circulating insulin and glucose with breast cancer risk: a Mendelian randomization analysis. Int J Epidemiol.

[256] Smith Byrne K, Appleby PN, Key TJ, Holmes MV, Fensom GK, Agudo A, Ardanaz E, Boeing H, Bueno-de-Mesquita HB, Chirlaque MD (2019). The role of plasma microseminoprotein-beta in prostate cancer: an observational nested case-control and Mendelian randomization study in the European prospective investigation into cancer and nutrition. Ann Oncol.

[257] Srinivas N, Rachakonda S, Hielscher T, Calderazzo S, Rudnai P, Gurzau E, Koppova K, Fletcher T, Kumar R (2019). Telomere length, arsenic exposure and risk of basal cell carcinoma of skin. Carcinogenesis.

[258] Takahashi H, Cornish AJ, Sud A, Law PJ, Disney-Hogg L, Calvocoressi L, Lu L, Hansen HM, Smirnov I, Walsh KM (2019). Mendelian randomization provides support for obesity as a risk factor for meningioma. Sci Rep.

[259] Wang X, Dai JY, Albanes D, Arndt V, Berndt SI, Bézieau S, Brenner H, Buchanan DD, Butterbach K, Caan B (2019). Mendelian randomization analysis of C-reactive protein on colorectal cancer risk. Int J Epidemiol.

[260] Yang H, Dai H, Li L, Wang X, Wang P, Song F, Zhang B, Chen K (2019). Age at menarche and epithelial ovarian cancer risk: a meta-analysis and Mendelian randomization study. Cancer Med.

[261] Yarmolinsky J, Relton CL, Lophatananon A, Muir K, Menon U, Gentry-Maharaj A, Walther A, Zheng J, Fasching P, Zheng W (2019). Appraising the role of previously reported risk factors in epithelial ovarian cancer risk: a Mendelian randomization analysis. PLoS Med.

[262] Zhou H, Zhang Y, Liu J, Yang Y, Fang W, Hong S, Chen G, Zhao S, Zhang Z, Shen J (2019). Education and lung cancer: a Mendelian randomization study. Int J Epidemiol.

[263] Zhu Y, Wei Y, Zhang R, Dong X, Shen S, Zhao Y, Bai J, Albanes D, Caporaso NE, Landi MT (2019). Elevated platelet count appears to be causally associated with increased risk of lung cancer: a Mendelian randomization analysis. Cancer Epidemiol Biomark Prev.

[264] Barahona Ponce C, Scherer D, Brinster R, Boekstegers F, Marcelain K, Gárate V, Müller B, de Toro G, Retamales J, Barajas O (2020). Gallstones, body mass index, C-reactive protein and gallbladder cancer - Mendelian randomization analysis of Chilean and European Genotype Data. Hepatology (Baltimore, Md).

[265] Baumeister SE, Leitzmann MF, Bahls M, Meisinger C, Amos CI, Hung RJ, Teumer A, Baurecht H (2020). Physical activity does not lower the risk of lung cancer. Cancer Res.

[266] Beeghly-Fadiel A, Khankari NK, Delahanty RJ, Shu XO, Lu Y, Schmidt MK, Bolla MK, Michailidou K, Wang Q, Dennis J (2020). A Mendelian randomization analysis of circulating lipid traits and breast cancer risk. Int J Epidemiol.

[267] Cheng WW, Wang ZK, Shangguan HF, Zhu Q, Zhang HY (2020). Are vitamins relevant to cancer risks? A Mendelian randomization investigation. Nutrition (Burbank, Los Angeles County, Calif).

[268] Cornish AJ, Law PJ, Timofeeva M, Palin K, Farrington SM, Palles C, Jenkins MA, Casey G, Brenner H, Chang-Claude J (2020). Modifiable pathways for colorectal cancer: a mendelian randomisation analysis. Lancet Gastroenterol Hepatol.

[269] Dusingize JC, Olsen CM, An J, Pandeya N, Law MH, Thompson BS, Goldstein AM, Iles MM, Webb PM, Neale RE (2020). Body mass index and height and risk of cutaneous melanoma: Mendelian randomization analyses. Int J Epidemiol.

[270] Fussey JM, Beaumont RN, Wood AR, Vaidya B, Smith J, Tyrrell J (2020). Does obesity cause thyroid cancer? A Mendelian randomization study. J Clin Endocrinol Metabolism.

[271] Gao XL, Jia ZM, Zhao FF, An DD, Wang B, Cheng EJ, Chen Y, Gong JN, Liu D, Huang YQ (2020). Obstructive sleep apnea syndrome and causal relationship with female breast cancer: a Mendelian randomization study. Aging.

[272] Ghoneim DH, Zhu J, Zheng W, Long J, Murff HJ, Ye F, Setiawan VW, Wilkens LR, Khankari NK, Haycock P (2020). Mendelian randomization analysis of n-6 polyunsaturated fatty acid levels and pancreatic cancer risk. Cancer Epidemiol Biomark Prev.

[273] Goto A, Yamaji T, Sawada N, Momozawa Y, Kamatani Y, Kubo M, Shimazu T, Inoue M, Noda M, Tsugane S (2020). Diabetes and cancer risk: a Mendelian randomization study. Int J Cancer.

[274] Guo Y, Lu Y, Jin H (2020). Appraising the role of circulating concentrations of micro-nutrients in epithelial ovarian cancer risk: a Mendelian randomization analysis. Sci Rep.

[275] Horsfall LJ, Burgess S, Hall I, Nazareth I (2020). Genetically raised serum bilirubin levels and lung cancer: a cohort study and Mendelian randomisation using UK Biobank. Thorax.

[276] Howe LJ, Hemani G, Lesseur C, Gaborieau V, Ludwig KU, Mangold E, Brennan P, Ness AR, St Pourcain B, Davey Smith G (2020). Evaluating shared genetic influences on nonsyndromic cleft lip/palate and oropharyngeal neoplasms. Genet Epidemiol.

[277] Howell AE, Robinson JW, Wootton RE, McAleenan A, Tsavachidis S, Ostrom QT, Bondy M, Armstrong G, Relton C, Haycock P (2020). Testing for causality between systematically identified risk factors and glioma: a Mendelian randomization study. BMC Cancer.

[278] Jiang X, Dimou NL, Zhu Z, Bonilla C, Lewis SJ, Lindström S, Kraft P, Tsilidis KK, Martin RM (2020). Allergy, asthma, and the risk of breast and prostate cancer: a Mendelian randomization study. Cancer Causes Control : CCC.

[279] Johnson KE, Siewert KM, Klarin D, Damrauer SM, Chang KM, Tsao PS, Assimes TL, Maxwell KN, Voight BF (2020). The relationship between circulating lipids and breast cancer risk: a Mendelian randomization study. PLoS Med.

[280] Jung SY, Papp JC, Sobel EM, Zhang ZF (2020). Mendelian randomization study: the association between metabolic pathways and colorectal cancer risk. Front Oncol.

[281] Kachuri L, Johansson M, Rashkin SR, Graff RE, Bossé Y, Manem V, Caporaso NE, Landi MT, Christiani DC, Vineis P (2020). Immune-mediated genetic pathways resulting in pulmonary function impairment increase lung cancer susceptibility. Nat Commun.

[282] Kazmi N, Haycock P, Tsilidis K, Lynch BM, Truong T, Martin RM, Lewis SJ (2020). Appraising causal relationships of dietary, nutritional and physical-activity exposures with overall and aggressive prostate cancer: two-sample Mendelian-randomization study based on 79148 prostate-cancer cases and 61106 controls. Int J Epidemiol.

[283] Khankari NK, Banbury BL, Borges MC, Haycock P, Albanes D, Arndt V, Berndt SI, Bézieau S, Brenner H, Campbell PT (2020). Mendelian randomization of circulating polyunsaturated fatty acids and colorectal cancer risk. Cancer Epidemiol Biomark Prev.

[284] King C, Mulugeta A, Nabi F, Walton R, Zhou A, Hyppönen E (2020). Mendelian randomization case-control PheWAS in UK Biobank shows evidence of causality for smoking intensity in 28 distinct clinical conditions. EClinicalMedicine.

[285] Kleinstern G, Camp NJ, Berndt SI, Birmann BM, Nieters A, Bracci PM, McKay JD, Ghesquières H, Lan Q, Hjalgrim H (2020). Lipid trait variants and the risk of non-Hodgkin lymphoma subtypes: a Mendelian randomization study. Cancer Epidemiol Biomark Prev.

[286] Larsson SC, Carter P, Kar S, Vithayathil M, Mason AM, Michaëlsson K, Burgess S (2020). Smoking, alcohol consumption, and cancer: a mendelian randomisation study in UK Biobank and international genetic consortia participants. PLoS Med.

[287] Larsson SC, Carter P, Vithayathil M, Kar S, Mason AM, Burgess S (2020). Insulin-like growth factor-1 and site-specific cancers: a Mendelian randomization study. Cancer Med.

[288] Li C, Stoma S, Lotta LA, Warner S, Albrecht E, Allione A, Arp PP, Broer L, Buxton JL (2020). Da Silva Couto Alves A et al: Genome-wide association analysis in humans links nucleotide metabolism to leukocyte telomere length. Am J Hum Genet.

[289] Li S, Xu Y, Zhang Y, Nie L, Ma Z, Ma L, Fang X, Ma X (2020). Mendelian randomization analyses of genetically predicted circulating levels of cytokines with risk of breast cancer. NPJ precision oncology.

[290] Liyanage UE, Law MH, Barrett JH, Iles MM, MacGregor S (2020). Is there a causal relationship between vitamin D and melanoma risk? A Mendelian randomization study. Br J Dermatol.

[291] Lu Y, Gentiluomo M, Lorenzo-Bermejo J, Morelli L, Obazee O, Campa D, Canzian F (2020). Mendelian randomisation study of the effects of known and putative risk factors on pancreatic cancer. J Med Genet.

[292] Luo S, Schooling CM, Wong ICK, Au Yeung SL (2020). Evaluating the impact of AMPK activation, a target of metformin, on risk of cardiovascular diseases and cancer in the UK Biobank: a Mendelian randomisation study. Diabetologia.

[293] Masuda T, Ogawa K, Kamatani Y, Murakami Y, Kimura T, Okada Y (2020). A Mendelian randomization study identified obesity as a causal risk factor of uterine endometrial cancer in Japanese. Cancer Sci.

[294] Murphy N, Carreras-Torres R, Song M, Chan AT, Martin RM, Papadimitriou N, Dimou N, Tsilidis KK, Banbury B, Bradbury KE (2020). Circulating levels of insulin-like growth factor 1 and insulin-like growth factor binding protein 3 associate with risk of colorectal cancer based on serologic and Mendelian randomization analyses. Gastroenterology.

[295] Murphy N, Knuppel A, Papadimitriou N, Martin RM, Tsilidis KK, Smith-Byrne K, Fensom G, Perez-Cornago A, Travis RC, Key TJ (2020). Insulin-like growth factor-1, insulin-like growth factor-binding protein-3, and breast cancer risk: observational and Mendelian randomization analyses with ∼430000 women. Ann Oncol.

[296] Papadimitriou N, Dimou N, Tsilidis KK, Banbury B, Martin RM, Lewis SJ, Kazmi N, Robinson TM, Albanes D, Aleksandrova K (2020). Physical activity and risks of breast and colorectal cancer: a Mendelian randomisation analysis. Nat Commun.

[297] Pedersen KM, Çolak Y, Ellervik C, Hasselbalch HC, Bojesen SE, Nordestgaard BG (2020). Loss-of-function polymorphism in IL6R reduces risk of JAK2V617F somatic mutation and myeloproliferative neoplasm: a Mendelian randomization study. EClinicalMedicine.

[298] Peng H, Wu X, Wen Y, Li C, Lin J, Li J, Xiong S, Zhong R, Liang H, Cheng B (2020). Association between systemic sclerosis and risk of lung cancer: results from a pool of cohort studies and Mendelian randomization analysis. Autoimmun Rev.

[299] Richardson TG, Sanderson E, Elsworth B, Tilling K, Davey Smith G. Use of genetic variation to separate the effects of early and later life adiposity on disease risk: Mendelian randomisation study. BMJ (Clin Res ed). 2020:369, m1203. 10.1136/bmj.m1203 PMC7201936 32376654

[300] Robinson T, Martin RM, Yarmolinsky J (2020). Mendelian randomisation analysis of circulating adipokines and C-reactive protein on breast cancer risk. Int J Cancer.

[301] Ruth KS, Day FR, Tyrrell J, Thompson DJ, Wood AR, Mahajan A, Beaumont RN, Wittemans L, Martin S, Busch AS (2020). Using human genetics to understand the disease impacts of testosterone in men and women. Nat Med.

[302] Saunders CN, Cornish AJ, Kinnersley B, Law PJ, Claus EB, Il'yasova D, Schildkraut J, Barnholtz-Sloan JS, Olson SH, Bernstein JL (2020). Lack of association between modifiable exposures and glioma risk: a Mendelian randomization analysis. Neuro-oncology.

[303] Seyed Khoei N, Jenab M, Murphy N, Banbury BL, Carreras-Torres R, Viallon V, Kühn T, Bueno-de-Mesquita B, Aleksandrova K, Cross AJ (2020). Circulating bilirubin levels and risk of colorectal cancer: serological and Mendelian randomization analyses. BMC Med.

[304] Sun X, Ye D, Du L, Qian Y, Jiang X, Mao Y (2020). Genetically predicted levels of circulating cytokines and prostate cancer risk: A Mendelian randomization study. Int J Cancer.

[305] Wang T, Ren C, Ni J, Ding H, Qi Q, Yan C, Deng B, Dai J, Li G, Ding Y (2020). Genetic association of plasma homocysteine levels with gastric cancer risk: a two-sample Mendelian randomization study. Cancer Epidemiol Biomark Prev.

[306] Wen Y, Wu X, Peng H, Li C, Jiang Y, Liang H, Zhong R, Liu J, He J, Liang W (2020). Cancer risks in patients with vitiligo: a Mendelian randomization study. J Cancer Res Clin Oncol.

[307] Went M, Cornish AJ, Law PJ, Kinnersley B, van Duin M, Weinhold N, Försti A, Hansson M, Sonneveld P, Goldschmidt H (2020). Search for multiple myeloma risk factors using Mendelian randomization. Blood Adv.

[308] Wong JYY, Zhang H, Hsiung CA, Shiraishi K, Yu K, Matsuo K, Wong MP, Hong YC, Wang J, Seow WJ (2020). Tuberculosis infection and lung adenocarcinoma: Mendelian randomization and pathway analysis of genome-wide association study data from never-smoking Asian women. Genomics.

[309] Yarmolinsky J, Bull CJ, Vincent EE, Robinson J, Walther A, Smith GD, Lewis SJ, Relton CL, Martin RM (2020). Association between genetically proxied inhibition of HMG-CoA reductase and epithelial ovarian cancer. Jama.

[310] Yuan S, Carter P, Bruzelius M, Vithayathil M, Kar S, Mason AM, Lin A, Burgess S, Larsson SC (2020). Effects of tumour necrosis factor on cardiovascular disease and cancer: a two-sample Mendelian randomization study. EBioMedicine.

[311] Yuan S, Carter P, Vithayathil M, Kar S, Giovannucci E, Mason AM, et al. Iron status and cancer risk in UK Biobank: a two-sample Mendelian randomization study. Nutrients. 2020:12(2). 10.3390/nu12020526 PMC7071358 32092884

[312] Yuan S, Kar S, Carter P, Vithayathil M, Mason AM, Burgess S, Larsson SC (2020). Is type 2 diabetes causally associated with cancer risk? Evidence from a two-sample Mendelian randomization study. Diabetes.

[313] Yuan S, Kar S, Vithayathil M, Carter P, Mason AM, Burgess S, Larsson SC (2020). Causal associations of thyroid function and dysfunction with overall, breast and thyroid cancer: A two-sample Mendelian randomization study. Int J Cancer.

[314] Zhang C, Hansen HM, Semmes EC, Gonzalez-Maya J, Morimoto L, Wei Q, Eward WC, DeWitt SB, Hurst JH, Metayer C (2020). Common genetic variation and risk of osteosarcoma in a multi-ethnic pediatric and adolescent population. Bone.

[315] Zheng J, Haberland V, Baird D, Walker V, Haycock PC, Hurle MR, Gutteridge A, Erola P, Liu Y, Luo S (2020). Phenome-wide Mendelian randomization mapping the influence of the plasma proteome on complex diseases. Nat Genet.

[316] Zhou W, Brumpton B, Kabil O, Gudmundsson J, Thorleifsson G, Weinstock J, Zawistowski M, Nielsen JB, Chaker L, Medici M (2020). GWAS of thyroid stimulating hormone highlights pleiotropic effects and inverse association with thyroid cancer. Nat Commun.

[317] Zhu J, Jiang X, Niu Z (2020). Alcohol consumption and risk of breast and ovarian cancer: a Mendelian randomization study. Cancer Genet.

[318] Zhu J, Shu X, Guo X, Liu D, Bao J, Milne RL, Giles GG, Wu C, Du M, White E (2020). Associations between genetically predicted blood protein biomarkers and pancreatic cancer risk. Cancer Epidemiol Biomark Prev.

[319] Chen M, Xu Y, Xu J, Chancoco H, Gu J (2021). Leukocyte telomere length and bladder cancer risk: a large case-control study and Mendelian randomization analysis. Cancer Epidemiol Biomark Prev.

[320] Jiang Y, Su Z, Li C, Wang R, Wen Y, Liang H, He J, Liang W (2021). Association between the use of aspirin and risk of lung cancer: results from pooled cohorts and Mendelian randomization analyses. J Cancer Res Clin Oncol.

[321] Kho PF, Amant F, Annibali D, Ashton K, Attia J, Auer PL, Beckmann MW, Black A, Brinton L, Buchanan DD (2021). Mendelian randomization analyses suggest a role for cholesterol in the development of endometrial cancer. Int J Cancer.

[322] Molina-Montes E, Coscia C, Gómez-Rubio P, Fernández A, Boenink R, Rava M, Márquez M, Molero X, Löhr M, Sharp L (2021). Deciphering the complex interplay between pancreatic cancer, diabetes mellitus subtypes and obesity/BMI through causal inference and mediation analyses. Gut.

[323] Ong JS, Derks EM, Eriksson M, An J, Hwang LD, Easton DF, Pharoah PP, Berchuck A, Kelemen LE, Matsuo K (2021). Evaluating the role of alcohol consumption in breast and ovarian cancer susceptibility using population-based cohort studies and two-sample Mendelian randomization analyses. Int J Cancer.

[324] Papadimitriou N, Dimou N, Gill D, Tzoulaki I, Murphy N, Riboli E, Lewis SJ, Martin RM, Gunter MJ, Tsilidis KK (2021). Genetically predicted circulating concentrations of micronutrients and risk of breast cancer: a Mendelian randomization study. Int J Cancer.

[325] Titova OE, Michaëlsson K, Vithayathil M, Mason AM, Kar S, Burgess S, Larsson SC (2021). Sleep duration and risk of overall and 22 site-specific cancers: a Mendelian randomization study. Int J Cancer.

[326] Xian W, Shen J, Zhou H, Liu J, Zhang Y, Zhang Z, Zhou T, Hong S, Yang Y, Fang W (2021). Mendelian randomization study indicates lack of causal relationship between physical activity and lung cancer. J Cancer Res Clin Oncol.

[327] Ye Y, Yang H, Wang Y, Zhao H (2021). A comprehensive genetic and epidemiological association analysis of vitamin D with common diseases/traits in the UK Biobank. Genet Epidemiol.

[328] Zhou W, Liu G, Hung RJ, Haycock PC, Aldrich MC, Andrew AS, Arnold SM, Bickeböller H, Bojesen SE, Brennan P (2021). Causal relationships between body mass index, smoking and lung cancer: univariable and multivariable Mendelian randomization. Int J Cancer.

[329] Burgess S, Dudbridge F, Thompson SG (2016). Combining information on multiple instrumental variables in Mendelian randomization: comparison of allele score and summarized data methods. Stat Med.

[330] Kyrgiou M, Kalliala I, Markozannes G, Gunter MJ, Paraskevaidis E, Gabra H, et al. Adiposity and cancer at major anatomical sites: umbrella review of the literature. BMJ (Clin Res ed), 2017. 356:j477. 10.1136/bmj.j477 PMC5421437 28246088

[331] World Cancer Research Fund/ American Institute for Cancer Research.Continuous Update Project Expert Report 2018. Diet, nutrition, physical activity andbreast cancer. Available at https://dietandcancerreport.org.

[332] Rukh G, Ahmad S, Ericson U, Hindy G, Stocks T, Renström F, Almgren P, Nilsson PM, Melander O, Franks PW (2016). Inverse relationship between a genetic risk score of 31 BMI loci and weight change before and after reaching middle age. Int J Obesity.

[333] Hidayat K, Yang CM, Shi BM (2018). Body fatness at a young age, body fatness gain and risk of breast cancer: systematic review and meta-analysis of cohort studies. Obes Rev.

[334] Kalliala I, Markozannes G, Gunter MJ, Paraskevaidis E, Gabra H, Mitra A, Terzidou V, Bennett P, Martin-Hirsch P, Tsilidis KK (2017). Obesity and gynaecological and obstetric conditions: umbrella review of the literature. BMJ (Clin Res ed).

[335] World Cancer Research Fund/ American Institute for Cancer Research.Continuous Update Project Expert Report 2018. Diet, nutrition, physical activity and endometrial cancer. Available at https://dietandcancerreport.org.

[336] World Cancer Research Fund/ American Institute for Cancer Research.Continuous Update Project Expert Report 2018. Diet, nutrition, physical activity and kidney cancer. Available at dietandcancerreport.org.

[337] Liu X, Sun Q, Hou H, Zhu K, Wang Q, Liu H, Zhang Q, Ji L, Li D (2018). The association between BMI and kidney cancer risk: An updated dose-response meta-analysis in accordance with PRISMA guideline. Medicine.

[338] World Cancer Research Fund/ American Institute for Cancer Research.Continuous Update Project Expert Report 2018. Diet, nutrition, physical activity and ovarian cancer. Available at https://dietandcancerreport.org.

[339] World Cancer Research Fund/ American Institute for Cancer Research.Continuous Update Project Expert Report 2018. Diet, nutrition, physical activity and colorectal cancer. Available at https://dietandcancerreport.org.

[340] World Cancer Research Fund/ American Institute for Cancer Research.Continuous Update Project Expert Report 2018. Diet, nutrition, physical activity and lung cancer. Available at https://dietandcancerreport.org.

[341] Lauby-Secretan B, Scoccianti C, Loomis D, Grosse Y, Bianchini F, Straif K (2016). Body fatness and cancer--viewpoint of the IARC Working Group. N Engl J Med.

[342] Allen NE, Key TJ, Dossus L, Rinaldi S, Cust A, Lukanova A, Peeters PH, Onland-Moret NC, Lahmann PH, Berrino F (2008). Endogenous sex hormones and endometrial cancer risk in women in the European Prospective Investigation into Cancer and Nutrition (EPIC). Endocrine-related Cancer.

[343] Davis SR, Wahlin-Jacobsen S (2015). Testosterone in women--the clinical significance. Lancet Diabetes Endocrinol.

[344] Platet N, Cathiard AM, Gleizes M, Garcia M (2004). Estrogens and their receptors in breast cancer progression: a dual role in cancer proliferation and invasion. Crit Rev Oncol/Hematol.

[345] Yamamoto T, Kitawaki J, Urabe M, Honjo H, Tamura T, Noguchi T, Okada H, Sasaki H, Tada A, Terashima Y (1993). Estrogen productivity of endometrium and endometrial cancer tissue; influence of aromatase on proliferation of endometrial cancer cells. J Steroid Biochem Mol Biol.

[346] Kaaks R, Lukanova A, Kurzer MS (2002). Obesity, endogenous hormones, and endometrial cancer risk: a synthetic review. Cancer Epidemiol Biomark Prev.

[347] Wentzensen IM, Mirabello L, Pfeiffer RM, Savage SA (2011). The association of telomere length and cancer: a meta-analysis. Cancer Epidemiol Biomark Prev.

[348] Zhang X, Zhao Q, Zhu W, Liu T, Xie SH, Zhong LX, Cai YY, Li XN, Liang M, Chen W (2017). The association of telomere length in peripheral blood cells with cancer risk: a systematic review and meta-analysis of prospective studies. Cancer Epidemiol Biomark Prev.

[349] Smith L, Luchini C, Demurtas J, Soysal P, Stubbs B, Hamer M, Nottegar A, Lawlor RT, Lopez-Sanchez GF, Firth J (2019). Telomere length and health outcomes: An umbrella review of systematic reviews and meta-analyses of observational studies. Ageing Res Rev.

[350] Hanahan D, Weinberg RA (2011). Hallmarks of cancer: the next generation. Cell.

[351] Aviv A, Anderson JJ, Shay JW (2017). Mutations, cancer and the telomere length paradox. Trends Cancer.

[352] McNally EJ, Luncsford PJ, Armanios M (2019). Long telomeres and cancer risk: the price of cellular immortality. J Clin Investig.

[353] Ma HQ, Cui LH, Li CC, Yu Z, Piao JM (2016). Effects of serum triglycerides on prostate cancer and breast cancer risk: a meta-analysis of prospective studies. Nutr Cancer.

[354] Ni H, Liu H, Gao R (2015). Serum lipids and breast cancer risk: a meta-analysis of prospective cohort studies. PloS One.

[355] Touvier M, Fassier P, His M, Norat T, Chan DS, Blacher J, Hercberg S, Galan P, Druesne-Pecollo N, Latino-Martel P (2015). Cholesterol and breast cancer risk: a systematic review and meta-analysis of prospective studies. Br J Nutr.

[356] Seth D, Garmo H, Wigertz A, Holmberg L, Hammar N, Jungner I, Lambe M, Walldius G, Van Hemelrijck M (2012). Lipid profiles and the risk of endometrial cancer in the Swedish AMORIS study. Int J Mol Epidemiol Genet.

[357] Bjørge T, Stocks T, Lukanova A, Tretli S, Selmer R, Manjer J, Rapp K, Ulmer H, Almquist M, Concin H (2010). Metabolic syndrome and endometrial carcinoma. Am J Epidemiol.

[358] Trabert B, Wentzensen N, Felix AS, Yang HP, Sherman ME, Brinton LA (2015). Metabolic syndrome and risk of endometrial cancer in the united states: a study in the SEER-medicare linked database. Cancer Epidemiol Biomark Prev.

[359] Lindemann K, Vatten LJ, Ellstrøm-Engh M, Eskild A (2009). Serum lipids and endometrial cancer risk: results from the HUNT-II study. Int J Cancer.

[360] Esposito K, Chiodini P, Capuano A, Bellastella G, Maiorino MI, Giugliano D (2014). Metabolic syndrome and endometrial cancer: a meta-analysis. Endocrine.

[361] Liu Y, Qin A, Li T, Qin X, Li S (2014). Effect of statin on risk of gynecologic cancers: a meta-analysis of observational studies and randomized controlled trials. Gynecologic Oncol.

[362] Lin X, Lu L, Liu L, Wei S, He Y, Chang J, Lian X (2017). Blood lipids profile and lung cancer risk in a meta-analysis of prospective cohort studies. J Clin Lipidol.

[363] Butler LM, Perone Y, Dehairs J, Lupien LE, de Laat V, Talebi A, Loda M, Kinlaw WB, Swinnen JV (2020). Lipids and cancer: emerging roles in pathogenesis, diagnosis and therapeutic intervention. Adv Drug Deliv Rev.

[364] Lim JY, Kwan HY. Roles of lipids in cancer. In: Advances in Lipid Metabolism. edn: London: IntechOpen Limited; 2018.

[365] Long J, Zhang CJ, Zhu N, Du K, Yin YF, Tan X, Liao DF, Qin L (2018). Lipid metabolism and carcinogenesis, cancer development. Am J Cancer Res.

[366] Yang X, Wang J (2019). The role of metabolic syndrome in endometrial cancer: a review. Frontiers Oncol.

[367] Jeong GH, Lee KH, Kim JY, Eisenhut M, Kronbichler A, van der Vliet HJ, Hong SH, Shin JI, Gamerith G (2019). Effect of statin on cancer incidence: an umbrella systematic review and meta-analysis. J Clin Med.

