# Supplementary material for: Systematic review of Mendelian randomization studies on risk of cancer
Source: BMC Med. 2022 Feb 2;20:41. doi: 10.1186/s12916-022-02246-y (PMC8809022; doi:10.1186/s12916-022-02246-y)
Supplement: Supplementary file 1 — Additional file 1. Supplementary methods. [file 12916_2022_2246_MOESM1_ESM.docx]

**Systematic review of Mendelian randomization studies on risk of cancer**

## Search Strategy

We searched the Medline (via PubMed) and Scopus databases from inception to October 2020 for MR studies investigating the association of genetically predicted risk factors with risk of cancer development or mortality, using the algorithm “(Mendelian Randomization OR Mendelian Randomisation OR genetic instrumental variable OR genetic instrument) AND (cancer OR carcinoma OR tumour OR tumor OR malign* OR neoplasia OR neoplasm)”. The electronic search was complemented by reviewing the references of relevant reviews and the references of the included studies. Two authors (GM and DK) independently performed the screening of the titles, abstracts and full texts. Discrepancies were resolved by discussion with KKT until consensus was reached.

## Inclusion-Exclusion criteria

MR studies that evaluated the association of any genetically predicted risk factor with risk of cancer development or mortality were deemed eligible. We only considered full original publications, and conference abstracts, letters, commentaries, editorials, reviews, study proposals and theoretical papers were excluded. We also excluded: a) studies that did not state that MR or a genetic instrumental variable was used and b) studies that only evaluated marginal genetic associations, namely direct associations of the genetic IVs with cancer without incorporating the association of the IVs with the risk factors. Methodological studies using an application of MR only as an example, and studies that identified potential genetic instruments for future MR studies were also deemed ineligible. We did not include studies on cancer prognosis or cancer differentiation. Finally, we excluded studies that did not provide usable data, namely at least an effect size for the studied association and a corresponding measure of uncertainty.

## Data Extraction

From all the eligible studies the following information was retrieved: name of first author, year of publication, risk factor evaluated, type of cancer, number of subjects and number of cases for both the exposure and the outcome analyses, source of exposure and outcome populations, population ancestry (for both the exposure and the outcome analyses), number of genetic instruments, exclusion criteria applied for the genetic instruments (p-value threshold, threshold for linkage disequilibrium, biological relevance), power of the genetic instruments and the corresponding effect estimate (when available), percentage of variance explained by the instruments, MR design (one-sample or two-sample, based on whether the gene-exposure and gene-outcome associations were estimated on the same or different populations), the specific analytical approach used for the main analysis as defined by the original publication authors (if multiple analyses were presented but it was unclear which was the main MR analysis, we opted for the inverse-variance-weighted analyses, if available), unit of comparison, the effect metric assessed (Odds Ratio, Hazard Ratio, beta, etc.), the effect size and the corresponding 95% confidence interval (CI) and p-value, the Q-statistic for heterogeneity for the instrument and the accompanying I^2^ value. Since studies frequently assessed multiple risk factors and/or cancer outcomes, we also extracted information on any adjusted thresholds used for multiple testing and whether the reported associations survived these thresholds. We further extracted information on a number of sensitivity MR methods, whenever these were performed and reported, namely MR-Egger, weighted median (WM), MRPRESSO and multivariable MR (MVMR). The MR-Egger is useful for the detection of IV assumption violation and correction by a) testing for directional pleiotropy, and b) testing and estimating an association after correcting for directional pleiotropy. The WM approach provides a consistent estimate if up to 50% of the information is derived from invalid instruments. MRPRESSO in an approach that allows for the evaluation of horizontal pleiotropy in three basic axes: a) detection of outlying genetic variants, b) correction via outlier removal and c) testing for effect distortion after outlier removal. MVMR accounts for mutual pleiotropic effects of the SNPs across multiple risk factors. Specifically, for the MR-Egger we kept information on both the intercept and the slope, for MRPRESSO we kept the number of SNPs excluded as well as the pleiotropy-corrected effect estimates, while for the MVMR we kept the exposure(s) adjusted for and the corresponding effect estimates. All data in each study were extracted by one reviewer (GM, AK, OD, DK, XZ, LW) and independently double-checked by another (GM, AK, OD, DK). Disagreements were resolved by consensus.
